# Epigenetically upregulating TROP2 and SLFN11 enhances therapeutic efficacy of TROP2 antibody drug conjugate sacitizumab govitecan

**DOI:** 10.1038/s41523-023-00573-8

**Published:** 2023-08-11

**Authors:** Ming Zhao, Timothy P. DiPeri, Maria Gabriela Raso, Xiaofeng Zheng, Yasmeen Qamar Rizvi, Kurt W. Evans, Fei Yang, Argun Akcakanat, Marco Roberto Estecio, Debu Tripathy, Ecaterina E. Dumbrava, Senthil Damodaran, Funda Meric-Bernstam

**Affiliations:** 1 Department of Investigational Cancer Therapeutics, University of Texas, MD Anderson Cancer Center, Houston, TX USA; 2 Department of Surgical Oncology, University of Texas, MD Anderson Cancer Center, Houston, TX USA; 3 Department of Translational Molecular Pathology, University of Texas, MD Anderson Cancer Center, Houston, TX USA; 4 Department of Bioinformatics and Computational Biology, University of Texas, MD Anderson Cancer Center, Houston, TX USA; 5 Department of Epigenetic and Molecular Carcinogenesis, University of Texas, MD Anderson Cancer Center, Houston, TX USA; 6 Department of Breast Medical Oncology, University of Texas, MD Anderson Cancer Center, Houston, TX USA; 7 Department of Breast Surgical Oncology, University of Texas, MD Anderson Cancer Center, Houston, TX USA

**Keywords:** Breast cancer, Cancer therapy

## Abstract

TROP2 antibody drug conjugates (ADCs) are under active development. We seek to determine whether we can enhance activity of TROP2 ADCs by increasing TROP2 expression. In metaplastic breast cancers (MpBC), there is limited expression of TROP2, and downregulating transcription factor ZEB1 upregulates E-cad and TROP2, thus sensitizing cancers to TROP2 ADC sacituzumab govitecan (SG). Demethylating agent decitabine decreases DNA methyltransferase expression and TROP2 promoter methylation and subsequently increases TROP2 expression. Decitabine treatment as well as overexpression of TROP2 significantly enhance SG antitumor activity. Decitabine also increases SLFN11, a biomarker of topoisomerase 1 inhibitor (TOP1) sensitivity and is synergistic with SG which has a TOP1 payload, in TROP2-expressing SLFN11-low BC cells. In conclusion, TROP2 and SLFN11 expression can be epigenetically modulated and the combination of demethylating agent decitabine with TROP2 ADCs may represent a novel therapeutic approach for tumors with low TROP2 or SLFN11 expression.

## Introduction

TROP2 (trophoblast cell surface antigen 2) is a transmembrane glycoprotein encoded by the *TACSTD2* gene (tumor-associated calcium signal transducer 2)^1^. Mainly expressed in epithelial cells, TROP2 is composed of an extracellular domain, a single transmembrane domain, and an intracellular domain which functions as intracellular calcium signal transducer^2–6^. TROP2 is expressed in several malignancies, including breast, lung, and urothelial carcinomas^7,8^, and is being actively pursued as a target for antibody-drug conjugates (ADCs).

Sacituzumab govitecan (IMMU-132, Gilead/Immunomedics) is a TROP2-targeted ADC covalently linked to SN-38, an active metabolite of the topoisomerase I inhibitor irinotecan^9–11^. Preclinical studies have demonstrated antitumor efficacy of sacituzumab govitecan (SG) in several tumor types, including triple negative breast cancer (TNBC)^10,12,13^. In several clinical trials, SG demonstrated durable objective responses in patients with heavily pretreated metastatic TNBC^14,15^, in hormone receptor positive/HER2 negative breast cancer^16^, as well as efficacy signal in several other tumor types including endometrial cancer, prostate cancer and small cell lung cancer^17,18^. Sacituzumab govitecan received accelerated approval by the U.S. Food and Drug Administration (FDA) in 2020 for treatment of advanced or metastatic TNBC, with full approval in 2021^19,20^. The randomized phase 3 ASCENT trial demonstrated that progression-free survival (PFS) and overall survival (OS) were significantly improved with SG in comparison to physician’s choice chemotherapy among patients with metastatic TNBC^18,21^. The median PFS was 5.6 months (95% confidence interval [CI], 4.3 to 6.3) with SG and 1.7 months (95% CI, 1.5 to 2.6) with chemotherapy (hazard ratio for disease progression or death, 0.41; 95% CI, 0.32 to 0.52; *P* < 0.001). The median OS was 12.1 months (95% CI, 10.7 to 14.0) with sacituzumab govitecan and 6.7 months (95% CI, 5.8 to 7.7) with chemotherapy (hazard ratio for death, 0.48; 95% CI, 0.38 to 0.59; *P* < 0.001). SG was also recently approved for advanced or metastatic urothelial cancer^20^ and is being explored for several other cancer types. Further, several other TROP2-targeted agents such as datopotamab deruxtecan (Dato-DXd, Daiichi Sankyo/AstraZeneca) and SKB264 (KLUS Pharma) are now in development with promising signal of activity^22–27^. A remaining clinical challenge is determining how TROP2 expression impacts sensitivity to TROP2-targeted ADCs.

Determinants of antitumor efficacy with ADCs are complex, and many factors may influence sensitivity, including sensitivity to the ADC payload. Target expression is an important factor in the mechanism of action of ADCs. Preclinically, the antitumor effect of SG has been associated with TROP2 levels in TNBC cells^28^. Clinically, although SG is effective across different levels of TROP2 expression, the greatest therapeutic efficacy has been reported in TNBC patients with medium and high TROP2 expression levels: objective response rates of 44%, 38%, and 22% were observed in patients with high (H-Score>200), medium (H-Score 100-200) and low expression (H-Score <100), respectively^18,21^.

In this study, we seek to explore novel strategies that enhance efficacy of SG in TROP2-low expressing tumors by pharmacological regulation of TROP2 expression. Our hypothesis was that epigenetic modulation of TROP2 with demethylating agents may enhance sensitivity to TROP2-targeted ADCs in TROP2-low expressing tumors.

## Results

### TROP2 protein and *TACSTD2* mRNA expression are low in metaplastic TNBC

**Fig. 1:**
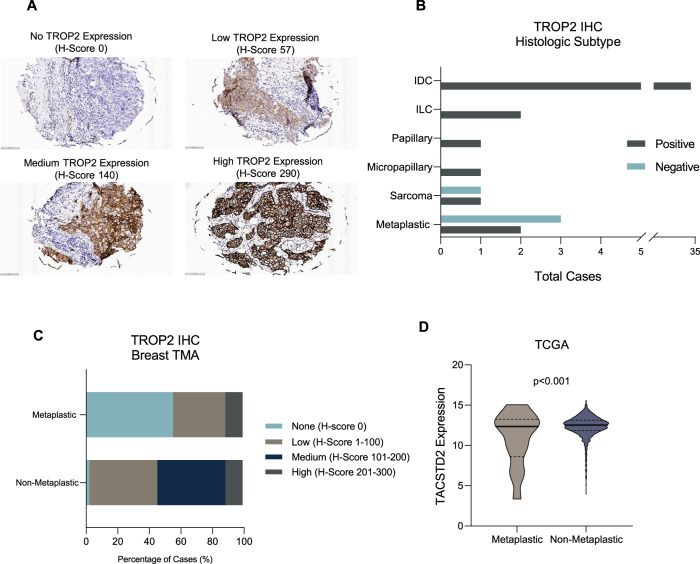
TROP2 and *TACSTD2* expression in breast cancer. **A** Breast cancer FFPE sections were assessed for TROP2 expression by IHC. Representative images demonstrate no TROP2expression (top left, H-score 0), low TROP2 expression (top right, H-score 57), medium TROP2 expression (bottom left, H-score 140), and high TROP2 expression (bottom right, H-score 290). **B** TROP2 positivity in breast cancer when stratified by histologic subtype. Of 41 cases evaluated, only 2 of 5 (40%) of metaplastic breast cases were TROP2 positive. **C** TROP2 expression in TMA of 106 breast tumors. Of the 97 non-metaplastic cases, 2 (2.1%) were TROP2 negative, 42 had low TROP2 expression (43.3%), 42 had medium TROP2 expression, and 11 had high TROP2 expression. Of the 9 metaplastic cases, 5 (55.6%) had no TROP2 expression, 3 (33.3%) had low TROP2 expression, and 1 (11.1%) had high TROP2 expression. **D** Comparison of *TACSTD2* expression in metaplastic vs. non-metaplastic breast tumors in TCGA. Expression of *TACSTD2* was higher in non-metaplastic tumors (*p* < 0.001, t-test).

We first determined the expression of TROP2 in breast cancer with immunohistochemistry (IHC) on surgically resected breast tumors. Representative images in Fig. 1A demonstrate a broad range of TROP2 expression: no TROP2 expression (top left, H-score 0), low TROP2 expression (top right, H-score 57), medium TROP2 expression (bottom left, H-score 140), and high TROP2 expression (bottom right, H-score 290). Breast samples were then assessed with TROP2 IHC to determine rates of TROP2 positivity (defined as TROP2 H-score > 0) when stratified by histologic subtype (Fig. 1B). Of the 41 cases evaluated, all the non-metaplastic tumors had at least some TROP2 protein expression, while only 2 of 5 (40%) metaplastic breast tumors were TROP2 positive.

We then assessed a tissue microarray (TMA) of 106 breast tumors for TROP2 expression by IHC (Fig. 1C). Of the 97 non-metaplastic cases, 2 (2.1%) were TROP2-negative, 42 (43.3%) had low TROP2 expression, 42 had intermediate TROP2 expression, and 11 had high TROP2 expression. Of the 9 metaplastic cases, 5 (55.6%) wereTROP2-negative, 3 (33.3%) had low TROP2 expression, and only 1 (11.1%) had high TROP2 expression. In 92 of these tumors, we also were able to perform IHC for SLFN11. SLFN11 was expressed in 4 of 8 metaplastic tumors and 12 of 88 non-metaplastic tumors. Overall, of 92 tumors that had TROP2 as well as SLFN11 testing, only 13 patients had expression of both TROP2 and SLFN11, 73 tumors had TROP2 expression but did not express SLFN11, three expressed SLFN11 and not TROP2, and three tumors did not express either biomarker.

After establishing that TROP2 protein expression was present in almost all non-metaplastic breast cancer, but in very few metaplastic breast tumors, we then sought to compare *TACSTD2* gene expression in breast tumors sequenced in The Cancer Genome Atlas (TCGA). Of the 1100 breast cases available in the TCGA, there were 14 (1.3%) metaplastic and 1086 (98.7%) non-metaplastic cases which were assessed. Expression of *TACSTD2* was lower in metaplastic tumors with a wider range of expression (*p* < 0.001) (Fig. 1D).

### TACSTD2 expression is associated with epithelial subtype in breast cancer

**Fig. 2:**
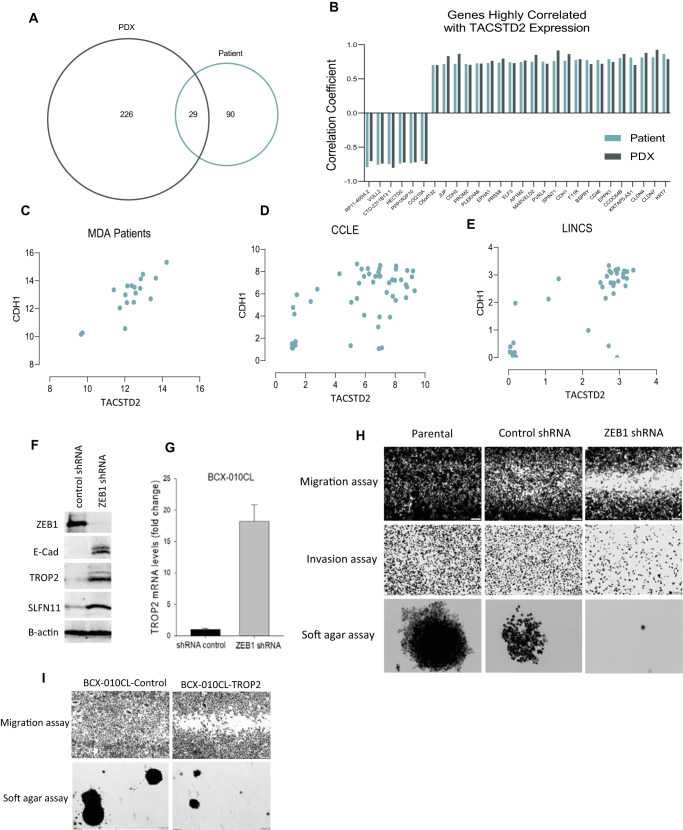
TROP2 and ZEB1 regulation of EMT in breast cancer cells. **A** Venn diagram of overlapping genes correlated with *TACSTD2* expression in patient and corresponding PDX samples. **B** Correlation of overlapping genes correlated with *TACSTD2* expression in patient and PDX samples. **C–E** Gene expression of *TACSTD2* was correlated with expression of CDH1 in breast tumors and cell lines. Correlation coefficient of *TACSTD2* vs. *CDH1* was 0.823 (*p* < 0.001, Pearson test) in breast tumors. Gene expression of *TACSTD2* was compared to expression of *CDH1* in breast cancer cell lines from the CCLE and LINCS. Correlation coefficient of *TACSTD2* vs. *CDH1* was 0.564 (*p* < 0.001, Pearson test) in the CCLE and 0.755 (*p* < 0.001, Pearson test) in the LINCS. **F** Immunoblotting of ZEB1 knockdown. Breast cancer BCX-010CL cells were transduced with lentivirus of *ZEB1* shRNA followed by puromycin selection. Cell lysates were subjected to SDS-PAGE and immunoblotted with antibodies against ZEB1, E-cad, SLFN11, and TROP2. **G** Quantitative PCR of TROP2 mRNA. Total RNA was extracted from ZEB1 knockdown and control cells, followed by reverse transcription. Quantitative real-time PCR was performed. Relative TROP2 mRNA was calculated with normalization by GAPDH (mean ± SEM). **H** Functional EMT assays of BCX**-**010CL cells with or without ZEB1 knockdown. Top row: Wound healing assay (migration assay). Cells seeded in 12-well plates were cultured to 100% confluency. A cross scratch was made on the cell layer followed by culture for 2 days to allow cell to migrate to close the scratch gap. Images of stained wells were taken. Middle row: Transwell assay (invasion assay). Cells seeded into transwell inserts invaded through basement membrane extract (BME) Matrigel in 24 h. The invaded and stained cells on the down-surface of the inserts were imaged. Low row: Soft agar assay (anchorage-independent growth assay). Cells seeded into 0.35% agar were cultured for 2-4 weeks to allow cell colony formation in the agar. The stained colonies were imaged. **I** EMT assays of BCX-010CL cells overexpressing TROP2. BCX-010CL cells were transfected with TROP2-expressing plasmid, followed by immunoblotting to verify TROP2 overexpression. Cells were subjected to wound-healing assay and soft agar assay to assess cell capability of migration and anchorage-independent growth, as described above. (Scale bars for 2H and 2I: 200 µm).

We first assessed expression of genes highly correlated with *TACSTD2* expression in breast tumors and corresponding patient-derived xenografts (PDXs) and found that 29 genes were overlapping between in 18 breast cancer samples and matching PDXs (Fig. 2A, B). Among these genes, TACSTD2 expression strongly correlated with expression of epithelial marker *CDH1* (E-Cadherin) (r^2^ = 0.823; *p* < 0.001) (Fig. 2C). Expression of *TACSTD2* was then compared to expression of *CDH1* in breast cancer cell lines from The Cancer Cell Line Encyclopedia (CCLE) (*N* = 51) and The Library of Integrated Network-Based Cellular Signatures (LINCS) (*N* = 35). There was also a significant correlation between *TACSTD2* and CDH1 in both CCLE (r^2^ = 0.564, *p* < 0.001) and LINCS (r^2^ = 0.755; *p* < 0.001) (Fig. 2D, E).

Given the correlation between *TACSTD2* and *CDH1* (Fig. 2C–E), we sought to determine whether TROP2 expression can be regulated with modulation of epithelial mesenchymal transition (EMT). As Zinc finger E-box binding homeobox 1 (ZEB1) is known to be one of the key transcriptional factors that control EMT process^29^, we examined if ZEB1 modulates expression of TROP2. The *ZEB1* mRNA was depleted in BCX-010CL breast cancer cells by shRNA knockdown (Fig. 2F). Immunoblotting showed that ZEB1 deficiency increased E-cadherin (E-cad) and TROP2 expression, compared to control cells (Fig. 2F). Quantitative real-time PCR demonstrated an increased *TACSTD2* mRNA level in ZEB1 depleted cells (Fig. 2G). Next, we examined the impact of ZEB1 deficiency on EMT in this breast cancer cell line. This was evidenced by (1) inhibited cell migration in wound healing migration assay; (2) reduced cell invasion capability in transwell Matrigel assay; and (3) decreased anchorage-independent cell growth in soft agar colony formation assay, compared to either shRNA control cells or parental cells (Fig. 2H). We also overexpressed TROP2 protein in BCX-010CL cells by plasmid transfection (Fig. 4N). We found that BCX-010CL cells overexpressing TROP2 displayed a more epithelial phenotype with decreased cell migration and anchorage-independent growth, compared to the control cells (Fig. 2I). Taken together these assays demonstrate that ZEB1 knockdown reversed EMT process and upregulated TROP2 mRNA and protein expression. We also knocked down ZEB1 in other breast cancer cell lines, including BCX-011CL and SUM159, and confirmed that ZEB1 deficiency increased TROP2 levels in the cells (Supplementary Figure 1A).

### Epigenetic modulators upregulate TROP2 expression in breast cancer cells

**Fig. 3:**
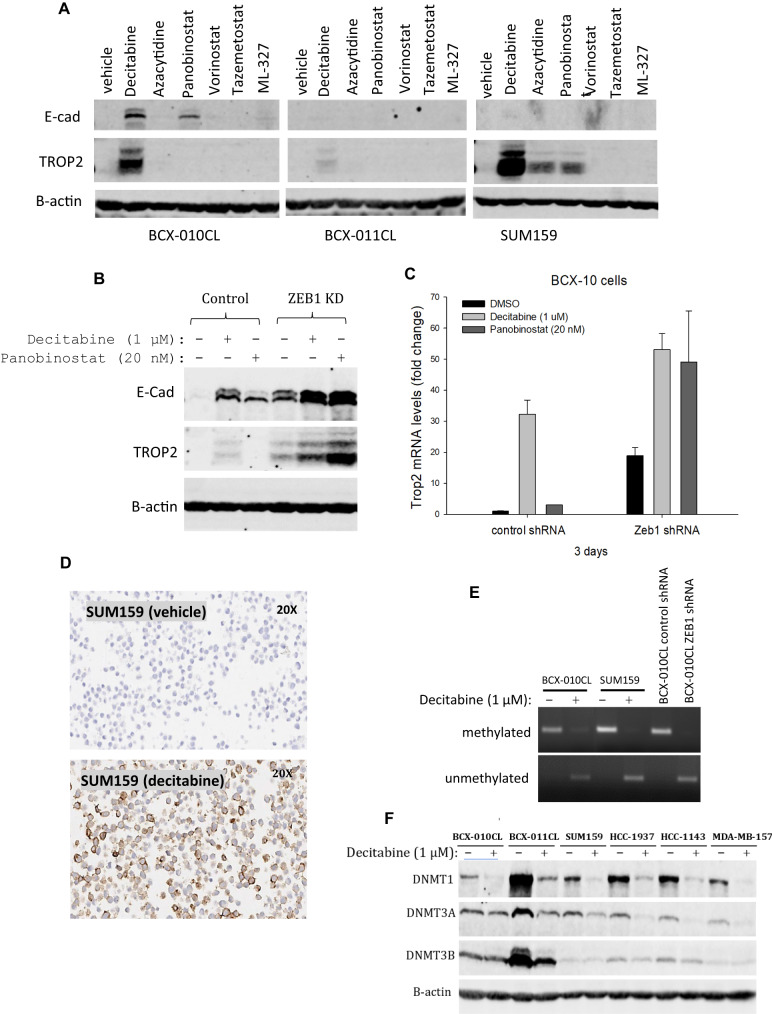
Epigenetic regulation of TROP2 expression in breast cancer cells. **A** Effects of epigenetic modulators on expression of E-cad and TROP2. Breast cancer cell lines BCX-010CL, BCX-011CL, and SUM159 cells were treated with decitabine, azacytidine, panobinostat, vorinostat, tazemetostat at 1, 1, 0.02, 1, 1, and 10 µM respectively for 3 days, followed by immunoblotting for E-cad and TROP2 with β-actin as control. **B**, **C** Effects of epigenetic modulators on expression of E-cad and TROP2 in ZEB1 knockdown cells. BCX-010CL cells with or without ZEB1 knockdown were treated with decitabine at 1 µM or panobinostat at 20 nM for 3 days. Protein levels of E-cad and TROP2 were determined by immunoblotting (B) and TROP2 mRNA levels were determined by quantitative PCR (C) (mean ± SEM). **D** Immunocytochemistry of TROP2. SUM159 cells were treated with decitabine at 1 µM for 3 days. After cell fixation, cell pellet blocks were processed and probed with anti-TROP2 antibody, followed by hematoxylin staining. **E** Methylation-specific PCR (MSP). Genomic DNA was extracted from BCX-010CL and BCX-011CL cells treated decitabine at 1 µM for 3 days, or from ZEB1 knockdown cells, and then subjected bisulfite CT conversion, followed by MSP using methylation specific primers specific to a CpG region in the TROP2 promoter. GAPDH was used as a control. **F** Immunoblotting of DNMTs. Breast cancer cell lines were treated with decitabine at 1 µM for 3 days. DNMT isoforms were detected by immunoblotting with antibodies against DNMT1, DNMT3A, and DNMT3B.

Considering the relevance of ZEB1 in epigenetic regulation, we next examined the effect of epigenetic modulators on TROP2 expression. We tested a panel of DNA methyltransferase (DNMT) inhibitors, histone deacetylase (HDAC) inhibitors, and EZH2 inhibitors in 6 breast cancer cell lines. We found that several DNMT and HDAC inhibitors enhanced expression of TROP2 and E-cadherin (E-cad) (Fig. 3A). Among these epigenetic modulators, the DNMT inhibitor decitabine was the most effective enhancer of TROP2 expression (Fig. 3A). Treatment with decitabine at 1 µM for 3 days substantially increased TROP2 levels in three breast cancer cell lines in which TROP2 basal levels were not detectable, particularly in cell line SUM159. Decitabine also increased E-cad expression in BCX-010CL cells. Next, we assessed the effect of decitabine on E-cad and TROP2 expression in ZEB1-deficient BCX-010CL cells. Immunoblotting showed that while decitabine increased E-cad and TROP2 levels in control cells, ZEB1 knockdown further tremendously boosted expression of both markers upon decitabine treatment (Fig. 3B). Similarly, the histone deacetylase inhibitor panobinostat-enhanced E-cad level was further elevated by ZEB1 knockdown. Interestingly, although panobibostat did not affect TROP2 expression in control cells, it clearly increased TROP2 levels in ZEB1-deficient cells (Fig. 3B). The effects of these epigenetic modulators and ZEB1 knockdown on TROP2 expression were also revealed at the mRNA levels (Fig. 3C). Immunocytochemistry confirmed that decitabine treatment increased TROP2 membrane expression in SUM159 cells (Fig. 3D). Promoter DNA methylation plays an important role in TROP2 transcription^30,31^. Our methylation-specific PCR (MSP) of defined CpG islands in *TROP2* promoter demonstrated that decitabine demethylated the TROP2 promoter in BCX-010CL and BCX-011Cl cells (Fig. 3E). Moreover, we found that ZEB1 deficiency was also able to abolish TROP2 promoter methylation (Fig. 3E). DNA methylation is mediated by DNMTs, and decitabine is known to inhibit DNMT enzymatic activity^32,33^. We found that decitabine was also able to reduce protein levels of all three DNMT isoforms across in BCX-010CL, BCX-011CL, and SUM159 cell lines, as well in other three breast cancer cell lines (Fig. 3F). This effect was also seen in other three breast cancer cell lines two of which have high TROP2 levels (Supplementary Fig. 1B, C). We also tested DNMT1-specific inhibitor GSK-3685032. Although GSK-3685032 decreased DNMT1 protein levels, it did not upregulate TROP2 expression in these breast cancer cell lines (Supplementary Fig. 1D).

### Upregulation of TROP2 expression enhances antitumor efficacy of sacituzumab govitecan

Our internalization results showed that SG binds to cell surface of TROP2-positive breast cancer cell lines, but not negative cell lines (Supplementary Fig. 2). The ADC drug bound on cell surface were internalized into the cells (Supplementary Figure 3).

**Fig. 4:**
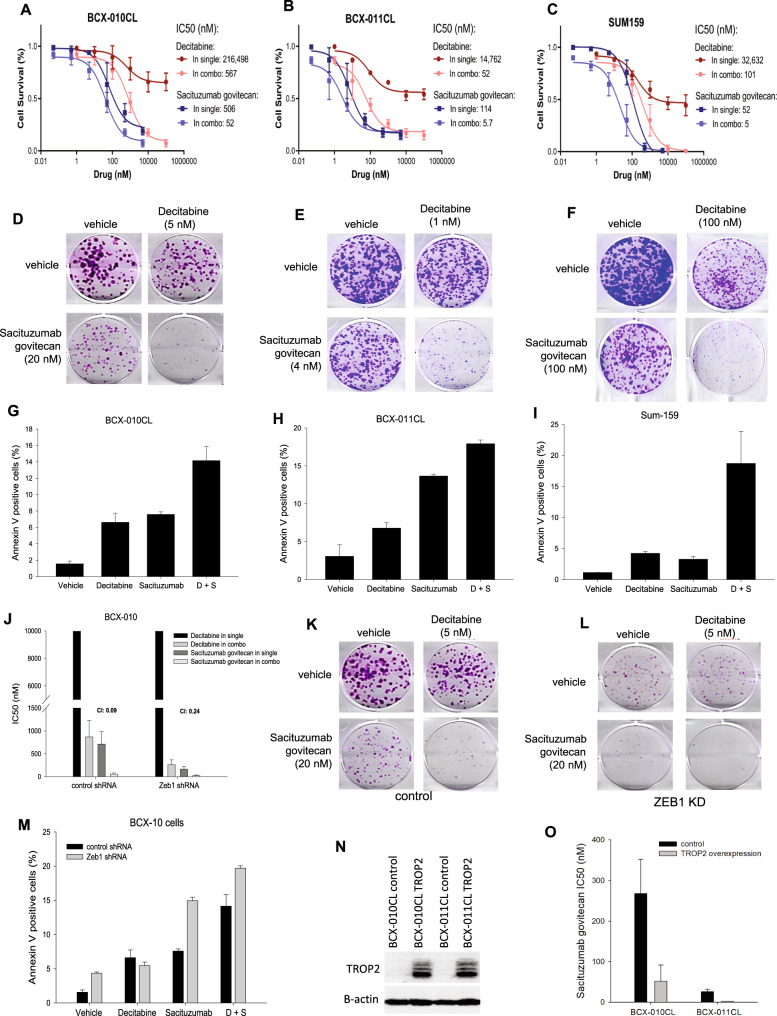
Synergistic antitumor efficacy of sacituzumab govitecan in combination with decitabine. **A–C** Effect of combination of SG and decitabine on cell survival. BCX-010CL, BCX-011CL, and SUM159 cells were treated with decitabine, SG or their combination at concentrations in serial dilutions for 3 days. After viability measurement, dose-response curves of cell inhibition were calculated. IC50s of drugs in single or combination groups and combination index (CI) were calculated using CalcuSyn. CI < 1.0 (curve left-shift): synergistic; CI = 1.0, additive; CI > 1.0 (curve right-shift): antagonistic). **D–F** Effect of combination on cell colony formation. BCX-010CL, BCX-011CL, and SUM159 cells were treated with decitabine, SG, or their combination at the indicated concentrations for 2-3 weeks. Crystal violet stained cell colonies were imaged. **G–I** Effect of combination on cell apoptosis. BCX-010CL, BCX-011CL, and SUM159 cells were treated with decitabine at 1 µM, SG at 0.1 µM, or their combination for 3 days. Annexin V staining positive cells were sorted by flow cytometry. Percentage of apoptotic cells was calculated by Annexin V positive cells over total cell populations. **J–M** Effect of ZEB1 knockdown on SG efficacy. In cell survival assay, shRNA control and ZEB1 shRNA cell lines were treated with decitabine, SG or their combination at concentrations in serial dilutions for 3 days. Drug IC50s and CI were calculated as described in Fig. 4A–F (**J**). In cell colony formation assay, the control and knockdown cells were treated with decitabine, SG, or their combination at the indicated concentrations for 3 weeks (K, L). In apoptosis assay, cells were treated with decitabine at 1 µM, SG at 0.1 µM, or their combination for 3 days. Annexin V positive apoptotic cells were measured by flow cytometry. Percentage of apoptotic cells over total cell populations was calculated (**M**). **N**, **O** Effect of TROP2 overexpression on SG. BCX-010CL cells were transfected with TROP2 expression plasmid. After G-418 selection, immunoblotting was performed to detect TROP2 expression (**N**). TROP2-overexpressing cells and control cells were treated with SG at concentrations in serial dilutions for 3 days. SG IC50 was calculated (**O**). Data in **A**, **B**, **C**, **G**, **H**, **I**, **J**, **M**, and **O** was presented by means ± SEM).

In this study, we determined whether pharmacologic upregulation of TROP2 expression enhances antitumor efficacy of SG in the three cell lines with low/no TROP2 expression. Results from cell viability assay showed that co-treatment with decitabine largely increased cell sensitivity to SG in all these cell lines (Fig. 4A–C). This was further confirmed by their synergistic combination index (CI) of 0.11, 0.12, and 0.11 in BCX-010CL, BCX-011CL, and SUM159 cell lines respectively (Supplementary Fig. 4A–C).

In colony formation assay, combination treatment with decitabine and SG exhibited greater inhibition efficacy on colony formation than single agent treatment (Fig. 4D–F, Supplementary Figure 4D–G). Bliss combination analysis of total colony areas demonstrated a remarkable combinatorial synergism between the drugs. We also evaluated the effect of these agents on cell apoptosis by flow cytometry analysis of Annexin V staining. Result showed that both decitabine and SG induced apoptosis as evidenced by increased Annexin V-positive cell percentage in all the three cell lines (Fig. 4G–I). Further enhanced cell apoptosis was also seen in the groups with combination treatment (Fig. 4G–I).

Next, we examined if increase of TROP2 expression by ZEB1 knockdown also sensitizes cells to SG. Cell viability examination of BCX-010CL cells showed that half maximal inhibitory concentration (IC50) of SG decreased in ZEB1 deficient cells compared to control cells (Fig. 4J). Combination of decitabine with SG showed enhanced therapeutic efficacy over either monotherapy in both control and ZEB1 knockdown cells with a CI of 0.09 and 0.24 respectively (Fig. 4J). Notably, ZEB1 knockdown substantially decreased cell capability to form cell colony in comparison to control BCX-010CL cells (Fig. 4K, L). Synergism of decitabine with SG on colony formation was also seen in both control and ZEB1 knockdown BCX-010CL cells (Fig. 4K, L, Supplementary Figure 4E, H). By Annexin V staining, we found that while ZEB1 deficiency did not change decitabine sensitivity, it significantly raised the percentage of apoptotic cells in both single and combination treatment with SG, compared to control cells (Fig. 4M).

In order to verify the impact of increase of TROP2 on cell sensitivity to SG, we overexpressed TROP2 in BCX-010CL and BCX-011CL cells (Fig. 4N). Immunofluorescence assay showed that when these BCX cells were incubated with SG ADC labeled with Alexa-Fluo-488, TROP2-overexpressing cells displayed strong immunofluorescent signal on cell membrane, compared to negative signal in control cells (Supplementary Figure 2). The immunofluorescent signal intensity in these TROP2-overexpressing cells was similar to that in MDA-MB-468 cells that express high levels of endogenous TROP2 (Supplementary Figure 3). Next, we demonstrated that overexpression of TROP2 reduced IC50 of SG in these cell lines (Fig. 4O).

### Cell signaling study identifies potential mechanisms for combination therapy with decitabine and sacituzumab govitecan

**Fig. 5:**
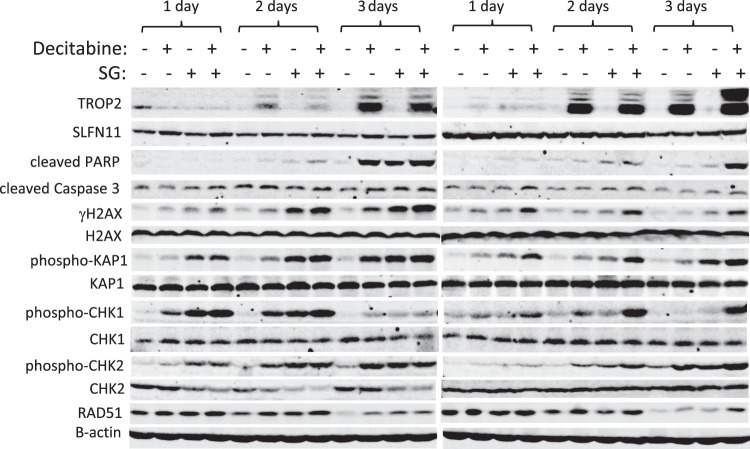
Effect of combination of decitabine and sacituzumab govitecan on cell signaling. BCX-010CL (left panel) and SUM159 (right panel) cells were treated with decitabine at 1 µM, sacituzumab govitecan at 0.1 µM, and their combination for 1, 2, 3 days. Cell lysates were subjected to SDS-PAGE, followed by immunoblotting using antibodies against cleaved PARP1, cleaved Caspase 3, TROP2, SLFN11, and RAD51, and also against phospho-KAP1, γH2AX, phospho-CHK1, phospho-CHK2, and their total proteins. Β-actin served as protein loading control.

Next, we determined the effect of decitabine and SG combination on cell signaling, aimed at exploring potential molecular mechanisms involved in this combinatorial therapy. BCX-010CL and SUM159 cells were treated with vehicle, or single agent decitabine, SG, or their combination for 1, 2, and 3 days. Result of immunoblotting assay showed that treatment with either decitabine or SG increased expression levels of phospho-KAP1, phospho-CHK2, γH2AX at all the three time points, all of which were further enhanced by combination treatments in both cell lines (Fig. 5). The drug combination also increased phospho-ATM and phospho-ATR at different times in the two cell lines (data not shown). These proteins are known to be key proteins in the DNA damage response (DDR) pathway. Furthermore, we found that combining decitabine and SG increased cleaved PARP1 levels over the levels induced by single agent treatment. (Fig. 5). In this time-course experiment, we noticed that decitabine boosted TROP2 expression from treatment day 2 with further increase by at day 3 in both cell lines, especially in SUM159 cell line (Fig. 5).

### Upregulation of SLFN11 expression sensitizes cells to sacituzumab govitecan and payload SN-38

**Fig. 6:**
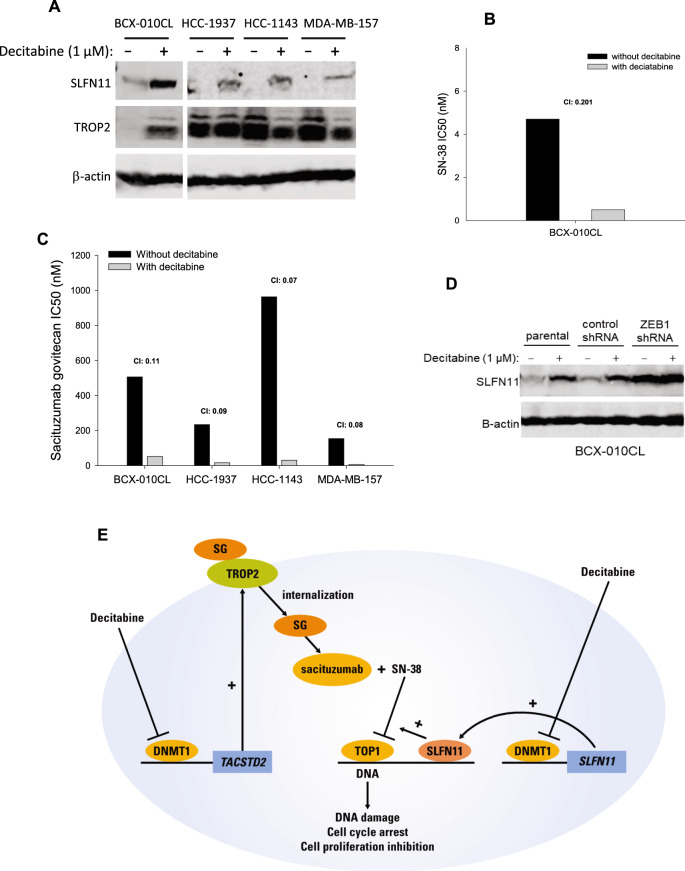
Effect of decitabine on SLFN11 expression. **A** Effect of decitabine on SLFN11 expression. Cell lines BCX-010CL, HCC-1937, HCC-1143, and MDA-MB-157 were treated with decitabine at 1 µM for 3 days. SLFN11 and TROP2 proteins in cell lysates were detected by immunoblotting. **B**, **C** Effect of decitabine on cell sensitivity to SN-38 (B) and SG (C). These breast cancer cell lines were treated with the indicated agents at concentrations in serial dilutions, with or without decitabine co-treatment for 3 days. IC50s of individual agents and combination index were calculated. **D** Effect of ZEB1 knockdown on SLFN11 expression in BCX-010CL cells. Cells with or without ZEB1 knockdown were treated with decitabine at 1 µM for 3 days, followed by immunoblotting for SLFN11. **E** Mechanism of decitabine action. In tumor cells, decitabine decreases promoter methylation of the *TACSTD2* gene by inhibiting DNA methyltransferase 1 (DNMT1), activating expression of *TACSTD2*/TROP2. On cell membrane, TROP2 protein binds to TROP2 ADC drug sacituzumab govitecan (SG). After internalization, SG is proteolytically cleaved in lysosome to release payload SN-38. SN-38 then moves into nucleus where it inhibits topoisomerase 1 (TOP1), leading to DNA damage, cell cycle arrest, and cell death. Decitabine also increases SLFN11 expression which further strengthens the chemocytoxic effect of SN-38 in the tumor cells.

Previous studies found that cell sensitivity to the TOP1 inhibitor SN-38 is associated with expression of Schlafen Family Member 11 (SLFN11) in solid tumors^34,35^. In this study, we sought to determine if SLFN11 plays a role in cell response to SG, which has a SN-38 payload, and if decitabine upregulates SLFN11 expression. First, we screened SLFN11 expression in a panel of 48 cell lines including 27 breast cancer cell lines (Supplementary Figure 5). We chose 3 TNBC cell lines (HCC-1937, HCC-1143, and MDA-MB-157) that do not express SLFN11, and a cell line (BCX-010CL) that expresses a low level of SLFN11. First, we determined the effect of decitabine on SLFN11 expression. Immunoblotting showed that treatment with decitabine for 3 days clearly enhanced SLFN11 expression in BCX-010CL cells and three TNBC cell lines, compared to vehicle controls (Fig. 6A). Next, we found that decitabine administration in these cell lines largely sensitized cells to treatments of TOP1 inhibitor SN-38 (ADC payload) as well as the ADC SG by substantially decreasing their IC50s (Fig. 6B, C). Combination index demonstrated to a strong synergism of therapeutic combination between decitabine and this TROP2 ADC and payloads (Fig. 6B, C). As expected, we also found that ZEB1 knockdown that increased TROP2 expression was also capable of increasing SLFN11 levels in BCX-010CL cells (Fig. 6D).

Given that TOP1 inhibitors have been demonstrated to affect DNA damage response (DDR) pathway^36^, we examined the effect of the combination of decitabine with SN-38 on DDR signaling in BCX-010CL and SUM159 cells. Immunoblotting results showed that while SN-38 itself increased the phosphorylation of KAP1, CHK1, CHK2, and γH2AX and expression of apoptosis marker cleaved PARP, combinatorial treatment further enhanced these changes (Supplementary Fig. 6). These influences on cell signaling are similar to what we found in the combination of decitabine with SG (Fig. 5), suggesting that decitabine may not only act by enhancing expression of TROP2, but also enhances activity of the TOP1 payload.

## Discussion

TROP2-targeted ADCs are emerging as a major therapeutic strategy, relevant for many cancer types. The efficacy of TROP2 ADCs in TNBC have been shown to be especially compelling, with improvements in overall survival documented with SG^15,21^. MpBC, a rare breast cancer subtype, has a worse prognosis and decreased survival compared to non-metaplastic TNBC^37^. There are currently no standardized treatment guidelines specifically for MpBC. In this study, we found that while almost all non-MpBC express TROP2, many MpBC are TROP2 negative. Further, we demonstrated that TROP2 expression can be epigenetically upregulated, providing a basis for combinatorial treatment in TROP2-targeting therapeutics.

The association of TROP2 and EMT has been controversial in the literature. While TROP2 is expressed in many epithelial cancers, there have also been publications suggesting that TROP2 promotes EMT^38,39^. Our data demonstrated a positive correlation between gene expression of *TACSTD2* and epithelial marker *CDH1*. We should note that although invasive lobular cancers often express TROP2, and yet loss of E-cadherin expression is a common feature of these carcinomas, that remain epithelial in nature^40^. Thus, our hypothesis is that TROP2 is usually expressed in epithelial cells, and we used E-cadherin regulation as an imperfect marker of epithelial state.

Transcription factor ZEB1 acts as a pivotal controller of EMT process^29,41^. In our breast cancer cell lines, we demonstrated that loss of ZEB1 reversed EMT and also substantially enhanced TROP2 expression. Previous studies reported that ZEB1 induced hypermethylation of the estrogen receptor α (ERα) promoter^42^ leading to silencing and this action was mediated by forming a protein complex with epigenetic regulators DNMT and HDAC^43^. This led us to assess whether epigenetic modulator agents could increase TROP2 expression.

In cell line screening, the DNMT inhibitor decitabine stood out among multiple epigenetic modulator agents including other DNMT inhibitors, HDAC inhibitors, and EZH2 inhibitors, as an effective enhancer of TROP2 expression. Other investigators have found that decitabine treatment increased TROP2 expression in lung cancer cell lines via promoter demethylation^44^. Although covalently binding to DMNTs and directly inhibiting DNMTs enzymatic activity is a primary mechanism of decitabine-induced demethylation, results from a recent study revealed that decitabine was also capable of inducing proteolytic degradation of DNMTs^45^. In our study, we demonstrated that decitabine decreased protein levels of three DNMT isoforms, DNMT1, DNMT3A, and DNMT3B, in the breast cell lines. This ubiquitination and lysosome-dependent degradation was previously found to be mediated through E3 ligase TNF receptor-associated factor 6 (TRAF6) in TNBC cells^45^.

Decitabine, and more recently its oral formulation in combination with cedazuridine (Inqovi, Astex/Taiho), have been approved for treatment of myelodysplastic syndromes (MDS)^46^. Decitabine is also being studied in the treatment of other cancers through multiple clinical trials^47^. Several preclinical and clinical studies in multiple tumor types have shown that decitabine-induced epigenetic reprogramming affects genome-wide methylation and expression profiling^45,48–52^.

Although for some ADCs, such as Folate Receptor alpha ADC mirvetixumab soravtansine^53^ and CEACAM5 ADC SAR408701^54^, efficacy has been shown to correlate with target expression in clinical trials, the role of TROP2 expression in antitumor activity of TROP2 ADCs has been more controversial. SG has been approved for metastatic TNBC, HR+, and urothelial cancers, and all three are tumor types with relatively frequent expression of TROP2. Preclinically, higher TROP2 expression correlated with SG efficacy, although SG also had growth inhibitory effect in tumors with low to moderate TROP2 expression^28^. In the ASCENT trial in TNBC, patients with higher TROP2 expression had a trend towards higher objective response rate and longer progression-free survival compared to those with lower expression, but SG led to improved oncologic outcomes compared to investigator treatment choice even in patients with lower TROP2 expression^18^. In the TROPiCs-02 trial in HR+ breast, antitumor activity was not dependent on TROP2 expression^55^. It is possible, this is at least in part because of the bystander effect of SG, that may decrease the importance of the amount of TROP2 expression. However, in a case series, lack of TROP2 expression and a novel mutation (T256R) in *TACSTD2* (gene encoding TROP2) that impairs TROP2 membrane localization have been reported as mechanisms of intrinsic and acquired resistance to SG respectively, suggesting a role of TROP2 expression and membrane localization for antitumor activity^56^.

In our study, we hypothesized that pharmacological upregulation of TROP2 expression in TNBC, especially in MpBC subtype, may produce significant therapeutic benefits in treatment with TROP2 ADCs. As a key finding of this study, we reported here that co-treatment of breast cancer cells with decitabine that increased TROP2 expression substantially enhanced cell sensitivity to SG, and the combination of these two agents led to a profound synergistic antitumor efficacy. These findings provide rational for pursuing decitabine as a combination partner to enhance the therapeutic efficacy of TROP2 ADCs.

Mechanistically, our data showed that combination treatment with decitabine and SG significantly enhanced their apoptotic efficacy compared to single agent treatment, as evidenced by Annexin V staining and increased cleaved PARP and caspase 3. Although there are many published studies that investigated involvement of TROP2 in multiple downstream signaling pathways, such as PKC, PI3K, and MAPK pathways^57–60^, few studies have reported on the role TROP2 in DNA damage response (DDR) pathway^61^. Here, we show that by targeting TROP2, combinatorial treatment of SG and decitabine activated the DDR pathway, as evidenced by elevated phosphoprotein levels of H2AX, CHK1, CHK2, and KAP1 compared to single agent treatment.

TOP1 inhibitors, such as irinotecan and its active metabolite SN-38, are chemotherapy agents which cause double strand DNA damage. In addition to inhibiting TOP1 activity, our data (Supplementary Figure 7) and other studies demonstrated that TOPO I inhibitors also reduce expression of the enzyme. Interestingly, we demonstrated robust synergy between decitabine and SG in not only BCX10CL and SUM159, where TROP2 was dramatically increased by decitabine, but also in BCX11CL where the TROP2 increase with decitabine treatment was a lot more modest (Fig. 3A), suggesting that there may be other mechanisms of synergy. Several studies have reported that sensitivity of cancer cells to these DNA-damaging agents is closely correlated with high SLFN11 levels^34,35,62,63^. Our results demonstrated that decitabine not only enhanced TROP2 expression, but also increased SLFN11 expression in these breast cancer cells. Therefore, the synergistic combination therapy of SG with decitabine could be explained by two separate pharmacological mechanisms: (1) decitabine increases TROP2 expression which is the target of TROP2 ADC sacituzumab govitecan; (2) decitabine also increases SLFN11 expression which sensitizes cells to TOP1 inhibitor payload SN-38. Figure 6E annotates these mechanisms.

In conclusion, we demonstrated that pharmacologic modulation of TROP2 and SLFN11 expression with decitabine led to enhanced efficacy of SG, a TROP2-targeted ADC, in MpBC models. We also, for the first time, provide a novel mechanism for decitabine-induced upregulation of TROP2 through epigenetic regulation. These findings provide supporting rational for pursuing decitabine as a combination partner for TROP2-targeted ADCs in TROP2 or SLFN11 low tumors.

## Materials and Methods

### TROP2 chromogenic immunohistochemistry

To assess TROP2 protein expression in breast cancer samples, a total of 42 formalin-fixed and paraffin embedded (FFPE) sections from surgically resected specimens were stained with anti-TROP2 antibody 1:2000 (clone ERP20043, Abcam, cat#214488), using an automated immunohistochemistry system (Leica Bond Rx, Leica Biosystems). Patients were enrolled on a study with prospective informed consent on a study that was approved by the Insitutitional Review Board (IRB) of the University of Texas MD Anderson Cancer Center. Additionally, two TMAs developed from breast cancer samples obtained by either image-guided biopsy or surgical resection were stained. These TMAs was generated with a protocol that is IRB approved with informed consent waiver. TROP2 membrane expression was evaluated and the percentage staining positivity (0-100%) and staining intensity (0 = no staining, 1 + = weak staining, 2 + = moderate staining, and 3 + = strong staining) were used to generate H-scores (0-300). TROP2 expression was classified as no TROP2 expression (H-score 0), low TROP2 expression (H-score 1-100), intermediate TROP2 expression (H-score 101-200), and high TROP2 expression (H-score 201-300). Tumors with no TROP2 expression (H-score 0) were considered TROP-2 negative, and tumors with any TROP2 expression (H-score 1-300) were considered TROP2 positive.

### RNA sequencing of breast tumors

Frozen fragments of breast tumors from patients (BCX P0) and corresponding breast cancer xenografts (BCX P1) were placed in lysis buffer, homogenized, and RNA was isolated using Norgen BIOTEK Total RNA Purification Plus Kit. RNA quality was assessed with an Agilent Bioanalyzer. Genomic RNA was quantified by Picogreen (Invitrogen) and quality was then assessed using 2200 Tapestation (Agilent). RNA was converted to doubled stranded complementary DNA (cDNA) using Ovation RNA-Seq System V2 Kit (Nugen). Libraries of cDNA were made using KAPA kits and capture was performed using Nimblegen whole exome V3 probes. Libraries were then sequenced on a HiSeq 2000 (Illumina) via a version 3 TruSeq paired-end flowcell per manufacturer’s instructions. FASTQ files are aligned to the reference genome (human Hg19) using STAR^64^, and HTSeq^65^ was used to obtain the read counts of genes. Qualities of raw reads were assessed using FastQC^66^. The read counts were normalized with DESeq2^67^.

### *TACTSD2* gene expression in breast tumors and cell lines

To assess expression of *TACSTD2* in metaplastic and non-metaplastic breast tumors in TCGA, expression data was downloaded from the cBio Portal “Breast Invasive Carcinoma” Firehouse Legacy data set (https://www.cbioportal.org/). Expression of *TACTSD2* was compared between metaplastic and non-metaplastic breast cancer samples. Correlation of *TACSTD2* expression in BCX P0 (patient) and BCX P1 (xenograft) were assessed. Relative gene expression of *TACSTD2* was then compared to expression of EMT regulatory genes in 18 breast tumors (BCX P0) and from breast cancer cell lines within two cancer cell line data repositories: CCLE (https://sites.broadinstitute.org/ccle/datasets) and LINCS (https://lincs.hms.harvard.edu/db/datasets/20348/main). Relative expression of *TACSTD2* was the compared to expression of *CDH1*. Analysis was carried out using log2 normalized counts after removing batch effect, and Spearman correlations were calculated.

### Cell lines, plasmids, drugs, and other reagents

Breast cancer cell lines MDA-BCX-010CL (BCX-010CL) and MDA-BCX-011CL (BCX-011CL) were generated from PDXs MDA-BCX-010 (BCX-010) and MDA-BCX-011 (BCX-011), both generated from metaplastic breast cancers. Breast cancer cell lines HCC-1937, HCC-1143, MDA-MB-157, and SUM159, were purchased from American Type Culture Collection (ATCC). Cells were cultured in Dulbecco’s modified Eagle’s medium/F-12 (DMEM) supplemented with 10% fetal bovine serum at 37° and humidified 5% CO_2_. Small hairpin RNA (shRNA) expression plasmids for *Zeb1* (TRCN0000017565 and TRCN0000235850) were purchased from Sigma-Aldrich. Expression plasmid pCMV6-entry-TROP2 was purchased from Origene. DNA methyltransferase inhibitors decitabine (5-Aza-2′-deoxycytidine, DAC), azacitidine (5-Azacytidine) and GSK-3685032; HDAC inhibitors panobinostat, vorinostat, belinostat; EZH2 inhibitor tazemetostat; and TOP1 inhibitor SN-38 were purchased from MedChemExpress (MCE). Sacituzumab govitecan was obtained from MD Anderson Cancer Center pharmacy. Anti-TROP2 antibody was purchased from Abcam. Antibodies against ZEB1, E-cadherin, cleaved PARP, cleaved Caspase 3, H2AX, γH2AX (Ser139), KAP1, phospho-KAP1 (Ser824), CHK1, phospho-CHK1 (Ser345), CHK2, phospho-CHK2 (Thr68), RAD51, SLFN11, DNMT1, DNMT3A, and DNMT3B were purchased from Cell Signaling Technology (CST). Anti-β-actin antibody (#A5441) was purchased from Sigma-Aldrich. Second antibodies Goat-anti-Rabbit-Alexa Fluor-680 (#A21076) and Goat-anti-Mouse- Dylight-800 (#610145-121) were purchased from Life Tech and Rockland Immunochemicals, respectively.

### ZEB1 shRNA knockdown

HEK-293 cells were seeded in 6-well plates at 3 ×10^5^ cells/well overnight. Cells were transfected with viral plasmids of *ZEB1* shRNAs using Lipofectamine 3000 Kit (ThermoFisher, #L3000001)). Lentivirus media were harvested at 48 h after transfection. BCX-010CL, BCX-011CL, and SUM159 cells were seeded in 6-well plates at 3 × 10^5^ cells/well overnight. Cells were infected with Zeb1 shRNA lentvirus for 2-3 days, followed by puromycin selection for 2-3 passages. ZEB1 western blotting (WB) was performed to select knockdown cells.

### Immunofluorescence of ADC binding on cell membrane

ADC drug SG was labeled with alexa fluorescent dye using Alexa Fluo 488 Conjugation Lightening Kit (Abcam, #ab236553), following the manufactory protocol. Final SG-Alexa-488 concentration was 1 µg/µl. To bind ADC to cells, cells were seeded on cover slides in 6-well plates overnight, followed by blocking with 3% BSA/PBS at room temperature for 1 h. Then cells were incubated with SG-Alexa-488 at 2 µg/ml at room temperature for 2 h, followed by PBS wash twice. For internalization assay, SG-Alexa-488 was added into the culture medium in the wells at 2 µg/ml for 3 h, and 24 h. Cells were fixed with 4% paraformaldehyde at room temperature for 15 min, PBS wash twice. The cover slices were mounted onto slide with DAPI counterstaining. Fluorescence on cell surface and in cytoplasm was monitored using Olympus BX51 fluorescent microscope and imaged using Nuance imaging system.

### Overexpression of TROP2

For TROP2 overexpression, breast cancer cells seeded in 6-well plates were transfected with TROP2 expression plasmid pCMV6-entry-TROP2 using Lipofectamine 3000 Kit. Two days after transfection, cells were treated with G-418 for 2-3 passages.

### Western blot analysis

Following treatments, cells were washed with cold PBS and lysed in 1x Laemmli buffer. The protein concentrations in the cell lysates were measured using Pierce BCA protein assay Kit (ThermoFisher, #23227). The same amount protein for each sample (20-50 µg/lane) was loaded to SDS-PAGE gel, followed by transferring proteins to a 0.2μm nitrocellulose membrane (Bio-Rad Laboratories). Membranes were blocked with blocking buffer Blocker Casein in PBS (ThermoFisher, #37582) at room temperature for 1 h, followed by immunoblotting with the primary antibodies in 5% BSA TBST buffer at 1:1000 dilution at room temperature overnight. After washing with TBST buffer, the immunoblotting membrane was then probed with the secondary antibodies with fluorescence conjugation at 1:10000 dilution. The immunoblots were visualized and the immunoblotting signal intensity quantitated using the Odyssey IR imaging system (Li-Cor Biosciences).

### Quantitative RT-PCR of TACSTD2

Following treatments, cells were lysed and total RNA was extracted using GeneJet RNA Purification Kit (ThermoFisher, #K0732) according to manufacturer’s manual. cDNA was synthesized from RNA using High-Capacity RNA-to-cDNA Kit (ThermoFisher, #4387406) following manufacturer’s manual. DNA amount in cDNA samples were quantitated using Qubity dsDNA Kit (ThermoFisher, #Q32584). Real time PCR was performed with the same amount DNA from each sample and TACSTD2 qPCR primer (ThermoFisher, Hs05055338_s1), using TaqMan Universal PCR Master Mix Kit (ThermoFisher, #4304437), under the PCR conditions: 50 °C, 2 min; 95 °C, 10 min; [95 °C, 15 s, 60 °C, 1 min] x 35 cycles. Fold changes of mRNA expression levels were calculated using formula 2^rrCT (rrCT: cycle threshold of treatment group – cycle threshold of control group).

### Immunocytochemistry

Formalin-fixed paraffin-embedded (FFPE) cell lines/tissue of 4-micron thickness slides were baked at 60 °C for 1 h. IHC staining was performed using Leica BondRXm autostainer (Leica Biosystems) for further processing. In brief, after deparaffinized by bond dewax solution, epitope retrieval (HIER) was performed with citrate buffer pH6, followed by washing steps. After peroxidase blocking (3% H_2_O_2_) for 20 min, slides were incubated in TROP2 rabbit monoclonal [EPR20043], catalog # ab214488 with a 1:2000 dilution for 15 min at ambient temperature. After transient washing, post-primary and polymer incubation for 8 min, followed by hematoxylin staining and wash steps. Afterwards, the slides were unloaded from autostainer, air dried and cover slipped with mounting media (Cytoseal XYL mounting media).

### Methylation-specific PCR (MSP)

Following treatments, cells were lysed and genomic DNA (gDNA) was extracted using Genomic DNA Purification Kit (ThermoFisher, #K5012) according to manufacturer’s manual. Bisulfite CT conversion of quantitated gDNA was performed using EZ DNA Methylation Kit (Zymo Research, #D5001) following manufacturer’s manual. MSP of TROP2 promoter CpG region was performed using PCR Supermix kit (ThermoFisher, #10572014) with the following primers: methylation forward: ATTTAAATATTAGTGGGG ACGGTC, methylation reverse: GAACCATAATAAAACGAAAAAACG; un-methylated forward: ATTTAAATATTAGTGGGGATGGTTG; and un-methylated reverse: CCAAACCATAATAAAACAAAAAAACA. PCR condition was set up as following: 94 °C, 1 min; [94 °C, 30 s, 62 °C, 30 s, 72 °C, 45 s] x 40 cycles; 72 °C, 2 min, 4 °C. About 200 bp PCR product was separated by 2% agarose gel.

### Cell viability assay

Cells were seeded in 96-well plates at densities of 0.15–0.6 × 10^4^ cells/100 μl per well in triplicates for each treatment dose. After adhering overnight, 100 μl of serially diluted drug solutions (decitabine, or sacituzumab govitecan, or their combination at ratio of 20:1) were added. Cells were incubated at 37 °C for 72 h. Cells were then fixed with 50% trichloroacetic (TCA) followed by staining with 0.4% sulforhodamine B (SRB) solution. OD values were read at 490 nm by plate reader Synergy 4 (BioTek). The half maximal inhibitory concentration (IC50) was determined using CalcuSyn software (Biosoft). Sigmoid drug-inhibition curves were made using GraphPad Prism v6.05 software. To evaluate combination efficacy, combination index (CI) was determined based on Chou-Talalay IC50 model and isobologram made using CalcuSyn. CI < 1.0 (curve left-shift): synergistic; CI = 1.0, additive; CI > 1.0 (curve right-shift): antagonistic.

### Colony formation assay

Cells were seeded in 6-well plates at a density of 1000 cells per well in triplicates for each treatment group. Next day, cells were treated with single drugs, or combination at the different concentrations. Culture medium was changed with fresh drugs twice a week. Cells were cultured for 3 weeks. Cell colonies were then fixed in 10% formalin and stained with 0.05% crystal violet in 25% methanol. The stained colonies were imaged and total colony area was quantitated using NIH ImageJ v.1.48 software. Effect-based Combination Index (CI) was calculated by inhibition percentage of single drug and combination treatments using Bliss combination model. CI = ((E_A_ + E_B_) – (E_A_*E_B_))/E_AB_, where E_A_, E_B_, and E_AB_ are effects of drug A, B and combination AB inhibition percentage. Here the effect is inhibition percentage of colony formation compared to vehicle controls. (CI < 1.0: synergistic; CI = 1.0: additive; CI > 1.0: antagonistic).

### Apoptosis assay

Cells were seeded in 6 cm plates at a density of 3 × 10^5^ cells per well in triplicates for each treatment group. The following day, cells were treated with decitabine and SG at 1 µM and 0.1 µM respectively, or their combination. After 72 h, floating and attached cells were collected. Using Annexin-V-FLUOS Staining Kit (Roche, # 11988549001), cells were stained with Annexin V fluorescence and propidium iodide (PI), following manufacturer’s protocol. Samples were analyzed by flow cytometry at The Flow Cytometry and Cellular Imaging Core Facility at MD Anderson Cancer Center. Percentage of Annexin V positive apoptotic cells were calculated.

### Cell migration assay

Wound healing assay was performed on ZEB1 knockdown cells. When cells cultured in 12-well plates were confluent, a cross scratch was made on the cell layer with 1 ml tip. Medium was changed to remove detached floating cells. Cells were cultured for 2 days to allow cell migration, followed by 10% formalin fixation and crystal violet staining. Photomicrograph of wound gap was performed, followed by quantitation using ImageJ. Relative fraction of wound gap was converted from wound gap area using a formula: wound gap area = (100 / %Area) x Total Area.

### Cell invasion assay

Transwell invasion assay was performed on Zeb1 knockdown cells. Transwell inserts (Corning, #354578) were coated with basement membrane extract (BME) Matrigel (Corning, #356234). Starved cells were seeded into the inserts at 5 × 10^4^ cells / insert in 0.3 ml serum-free medium. The inserts were assembled into wells of 24-well plates containing 0.5 ml medium with 10% FBS. Cells were cultured for 24 h. Cells on the up-surface of the inserts were removed by scrubbing with cotton swabs. Cells that invaded through the BME were fixed on the down-surface of the inserts with 10% formalin, followed by staining with 0.4% crystal violet. The stained cells were imaged by inverted microscope at x100 magnifications. Cells numbers were counted per image field. Nine fields were quantitated for each group.

### Anchorage-independent cell growth assay

Soft agar assay was performed on ZEB1 knockdown cells. 6-well plates were coated with 0.5% bottom agar (Difco Agar Noble, BD, #214220). Cells were mixed with 0.35% top agar containing 10% FBS and seeded onto the bottom agar wells at 5000 cells/well. 1 ml complete medium was added on top the agar. Cells were fed twice a week and cultured for 3 weeks. Colonies in the agar were stained with iodonitrotetrazolium chloride (INT) overnight, followed by colony photomicrograph.

### Xenograft generation

All animal implantations were approved by Institutional Animal Care and Use Committee (IACUC) of the University of Texas MD Anderson Cancer Center prior to being performed. All animals were under frequent veterinarian monitoring and animals had constant access to food and water as well bedding. Details of the generation of all the PDXs analyzed in this study has been previously published^68^. In short, surgical resected breast tumors were implanted into the flanks or fourth mammary fat pad of female immunodeficient mice (NOD.Cg-Prkdc^scid^ Il2rg^tm1Wjl^/SzJ; The Jackson Laboratory, Bar Harbor ME; or athymic nude mice). Surigcal implantation was done under isoflourane (vaporized) anesthesia and buprenorphine was given as preoperative analgestic. Once the implanted tumors grew to 1.5 cm in diameter, the animal were euthanized by carbon dioxide inhalation. Portions of the xenografts were passaged to additional mice as well as flash frozen or formalin-fixed, paraffin embedeed. First passage xenograft samples were used for RNASeq and TROP2 IHC. All patient samples were collected from consenting patients at the University of Texas MD Anderson Cancer Center under a protocol approved an Institutional Review Board.

### Statistical analysis

Student’s t-test was performed to compare two groups for patients’ and in vitro samples. Data were presented as means ± SEM. Pearson test was used for correlation between two groups for patients’ samples and cell line database.

### Reporting summary

Further information on research design is available in the Nature Research Reporting Summary linked to this article.

### Supplementary information

ARRIVE checklist

Supplementary Materials

Reporting Summary

## Data Availability

The RNA-seq data is scheduled for release in GEO on 23^rd^ August 2023 under the accession number GSE235812. This data and other data that support the findings of this study are available from the corresponding author, upon reasonable request.

## References

[1] Calabrese G (2001). Assignment of TACSTD1 (alias TROP1, M4S1) to human chromosome 2p21 and refinement of mapping of TACSTD2 (alias TROP2, M1S1) to human chromosome 1p32 by in situ hybridization. Cytogenet Cell Genet..

[2] El Sewedy T, Fornaro M, Alberti S (1998). Cloning of the murine TROP2 gene: conservation of a PIP2-binding sequence in the cytoplasmic domain of TROP-2. Int J. Cancer.

[3] Linnenbach AJ (1989). Sequence investigation of the major gastrointestinal tumor-associated antigen gene family, GA733. Proc. Natl. Acad. Sci. USA.

[4] Pavsic M, Ilc G, Vidmar T, Plavec J, Lenarcic B (2015). The cytosolic tail of the tumor marker protein Trop2–a structural switch triggered by phosphorylation. Sci. Rep..

[5] Vidmar T, Pavsic M, Lenarcic B (2013). Biochemical and preliminary X-ray characterization of the tumor-associated calcium signal transducer 2 (Trop2) ectodomain. Protein Expr. Purif..

[6] Ripani E, Sacchetti A, Corda D, Alberti S (1998). Human Trop-2 is a tumor-associated calcium signal transducer. Int J. Cancer.

[7] Shvartsur A, Bonavida B (2015). Trop2 and its overexpression in cancers: regulation and clinical/therapeutic implications. Genes Cancer.

[8] Lenart, S. et al. Trop2: Jack of All Trades, Master of None. *Cancers (Basel)* **12**, 10.3390/cancers12113328 (2020). 10.3390/cancers12113328 PMC7696911 33187148

[9] Goldenberg DM, Sharkey RM (2020). Sacituzumab govitecan, a novel, third-generation, antibody-drug conjugate (ADC) for cancer therapy. Expert Opin. Biol. Ther..

[10] Goldenberg DM, Cardillo TM, Govindan SV, Rossi EA, Sharkey RM (2015). Trop-2 is a novel target for solid cancer therapy with sacituzumab govitecan (IMMU-132), an antibody-drug conjugate (ADC). Oncotarget.

[11] Syed YY (2020). Sacituzumab govitecan: first approval. Drugs.

[12] Perrone E (2020). Preclinical activity of sacituzumab govitecan, an antibody-drug conjugate targeting trophoblast cell-surface antigen 2 (Trop-2) Linked to the Active Metabolite of Irinotecan (SN-38), in Ovarian Cancer. Front Oncol..

[13] Lopez S (2020). Preclinical activity of sacituzumab govitecan (IMMU-132) in uterine and ovarian carcinosarcomas. Oncotarget.

[14] Bardia A (2017). Efficacy and safety of anti-trop-2 antibody drug conjugate sacituzumab govitecan (IMMU-132) in heavily pretreated patients with metastatic triple-negative breast cancer. J. Clin. Oncol..

[15] Bardia A (2019). Sacituzumab Govitecan-hziy in Refractory Metastatic Triple-Negative Breast Cancer. N. Engl. J. Med..

[16] Kalinsky K (2020). Sacituzumab govitecan in previously treated hormone receptor-positive/HER2-negative metastatic breast cancer: final results from a phase I/II, single-arm, basket trial. Ann. Oncol..

[17] Tagawa ST (2021). TROPHY-U-01: A Phase II open-label study of sacituzumab govitecan in patients with metastatic urothelial carcinoma progressing after platinum-based chemotherapy and checkpoint inhibitors. J. Clin. Oncol..

[18] Bardia A (2021). Biomarker analyses in the phase III ASCENT study of sacituzumab govitecan versus chemotherapy in patients with metastatic triple-negative breast cancer. Ann. Oncol..

[19] FDA grants accelerated approval to sacituzumab govitecan-hziy for metastatic triple negative breast cancer. *News release. FDA. April 22*, (2020).

[20] FDA grants regular approval to sacituzumab govitecan for triple-negative breast cancer. *News release. FDA. April 7*, (2021).

[21] Bardia A (2021). Sacituzumab govitecan in metastatic triple-negative breast cancer. N. Engl. J. Med..

[22] Borghaei H (2021). TROPION-Lung04: Datopotamab Deruxtecan (Dato-DXd) Plus Durvalumab and Platinum-Based Chemotherapy in Advanced NSCLC. J. Thorac. Oncol..

[23] Garon EB (2021). TROPION-PanTumor01: Updated Results From the NSCLC Cohort of the Phase 1 Study of Datopotamab Deruxtecan in Solid Tumors. J. Thorac. Oncol..

[24] Johnson M (2021). A Phase 2 Study of Datopotamab Deruxtecan (Dato-DXd) in Advanced NSCLC With Actionable Genomic Alterations (TROPION-Lung05). J. Thorac. Oncol..

[25] Levy B (2021). TROPION-Lung02: Datopotamab Deruxtecan (Dato-DXd) Plus Pembrolizumab and Platinum-Based Chemotherapy in Advanced NSCLC. J. Thorac. Oncol..

[26] Garon EB (2021). Efficacy of datopotamab deruxtecan (Dato-DXd) in patients (pts) with advanced/metastatic (adv/met) nonsmall cell lung cancer (NSCLC) and actionable genomic alterations (AGAs): Preliminary results from the phase I TROPION-PanTumor01 study. Ann. Oncol..

[27] Rodon J (2021). An open-label, global, first-in-human study of SKB264 in patients with locally advanced or metastatic solid tumors. Ann. Oncol..

[28] Cardillo TM (2020). Predictive biomarkers for sacituzumab govitecan efficacy in Trop-2-expressing triple-negative breast cancer. Oncotarget.

[29] Taube JH (2010). Core epithelial-to-mesenchymal transition interactome gene-expression signature is associated with claudin-low and metaplastic breast cancer subtypes. Proc. Natl. Acad. Sci. USA.

[30] Sin STK (2018). Down-regulation of TROP-2 predicts poor prognosis of hepatocellular carcinoma patients. Hepatol. Commun..

[31] Zimmers SM (2018). TROP2 methylation and expression in tamoxifen-resistant breast cancer. Cancer Cell Int..

[32] Jabbour E, Issa JP, Garcia-Manero G, Kantarjian H (2008). Evolution of decitabine development: accomplishments, ongoing investigations, and future strategies. Cancer.

[33] Derissen EJ, Beijnen JH, Schellens JH (2013). Concise drug review: azacitidine and decitabine. Oncologist.

[34] Tian L (2014). Schlafen-11 sensitizes colorectal carcinoma cells to irinotecan. Anticancer Drugs.

[35] Shee K, Wells JD, Jiang A, Miller TW (2019). Integrated pan-cancer gene expression and drug sensitivity analysis reveals SLFN11 mRNA as a solid tumor biomarker predictive of sensitivity to DNA-damaging chemotherapy. PLoS One.

[36] Xu Y, Her C (2015). Inhibition of Topoisomerase (DNA) I (TOP1): DNA damage repair and anticancer therapy. Biomolecules.

[37] Nelson RA, Guye ML, Luu T, Lai LL (2015). Survival outcomes of metaplastic breast cancer patients: results from a US population-based analysis. Ann. Surg. Oncol..

[38] Wang J (2011). Loss of Trop2 promotes carcinogenesis and features of epithelial to mesenchymal transition in squamous cell carcinoma. Mol. Cancer Res..

[39] Zhao W (2018). Trop2 is a potential biomarker for the promotion of EMT in human breast cancer. Oncol. Rep..

[40] McCart Reed AE (2016). An epithelial to mesenchymal transition programme does not usually drive the phenotype of invasive lobular carcinomas. J. Pathol..

[41] Soundararajan R, Paranjape AN, Maity S, Aparicio A, Mani SA (2018). EMT, stemness and tumor plasticity in aggressive variant neuroendocrine prostate cancers. Biochim Biophys. Acta Rev. Cancer.

[42] Zhang J (2017). ZEB1 induces ER-alpha promoter hypermethylation and confers antiestrogen resistance in breast cancer. Cell Death Dis..

[43] Zhou C (2017). ZEB1 confers stem cell-like properties in breast cancer by targeting neurogenin-3. Oncotarget.

[44] Lin JC (2012). TROP2 is epigenetically inactivated and modulates IGF-1R signalling in lung adenocarcinoma. EMBO Mol. Med..

[45] Yu J (2018). DNA methyltransferase expression in triple-negative breast cancer predicts sensitivity to decitabine. J. Clin. Invest..

[46] NCI. Cancer Treatment/Cancer Drugs. https://www.cancer.gov/about-cancer/treatment/drugs/decitabine.

[47] NCI. Cancer Treatment/Clinical Trials. https://www.cancer.gov/about-cancer/treatment/clinical-trials/intervention/decitabine.

[48] Zhou S, Liu P, Zhang H (2017). Bioinformatic analysis of the effects and mechanisms of decitabine and cytarabine on acute myeloid leukemia. Mol. Med. Rep..

[49] Zhang Z (2020). Decitabine induces change of biological traits in myelodysplastic syndromes via FOXO1 activation. Front Genet.

[50] Dahn ML (2020). Decitabine response in breast cancer requires efficient drug processing and is not limited by multidrug resistance. Mol. Cancer Ther..

[51] Bhatla T (2012). Epigenetic reprogramming reverses the relapse-specific gene expression signature and restores chemosensitivity in childhood B-lymphoblastic leukemia. Blood.

[52] Al-Romaih K (2007). Modulation by decitabine of gene expression and growth of osteosarcoma U2OS cells in vitro and in xenografts: identification of apoptotic genes as targets for demethylation. Cancer Cell Int..

[53] Martin LP (2017). Characterization of folate receptor alpha (FRalpha) expression in archival tumor and biopsy samples from relapsed epithelial ovarian cancer patients: A phase I expansion study of the FRalpha-targeting antibody-drug conjugate mirvetuximab soravtansine. Gynecol. Oncol..

[54] Charles Ricordel, F. B. et al. Safety and efficacy of tusamitamab ravtansine (SAR408701) in long-term treated patients with nonsquamous non–small cell lung cancer (NSQ NSCLC) expressing carcinoembryonic antigen-related cell adhesion molecule 5 (CEACAM5). *J. Clin. Oncol.* **40** (2022).

[55] Hope Rugo, A. B. et al. Sacituzumab Govitecan (SG) vs Treatment of Physician’s Choice (TPC): Efficacy by Trop-2 Expression in the TROPiCS-02 Study of Patients (Pts) With HR+/HER2– Metastatic Breast Cancer (mBC) *SABCS*, GS1-11 (2022).

[56] Coates JT (2021). Parallel genomic alterations of antigen and payload targets mediate polyclonal acquired clinical resistance to sacituzumab govitecan in triple-negative breast cancer. Cancer Discov..

[57] Cubas R, Zhang S, Li M, Chen C, Yao Q (2010). Trop2 expression contributes to tumor pathogenesis by activating the ERK MAPK pathway. Mol. Cancer.

[58] Li X (2017). TROP2 promotes proliferation, migration and metastasis of gallbladder cancer cells by regulating PI3K/AKT pathway and inducing EMT. Oncotarget.

[59] Mori Y (2019). Trophoblast cell surface antigen 2 (Trop-2) phosphorylation by protein kinase C alpha/delta (PKCalpha/delta) enhances cell motility. J. Biol. Chem..

[60] Tang G (2019). TROP2 increases growth and metastasis of human oral squamous cell carcinoma through activation of the PI3K/Akt signaling pathway. Int J. Mol. Med..

[61] Hsu EC (2020). Trop2 is a driver of metastatic prostate cancer with neuroendocrine phenotype via PARP1. Proc. Natl. Acad. Sci. USA.

[62] Kang MH (2015). Activity of MM-398, nanoliposomal irinotecan (nal-IRI), in Ewing’s family tumor xenografts is associated with high exposure of tumor to drug and high SLFN11 expression. Clin. Cancer Res.

[63] Luan J, Gao X, Hu F, Zhang Y, Gou X (2020). SLFN11 is a general target for enhancing the sensitivity of cancer to chemotherapy (DNA-damaging agents. J Drug Target..

[64] Dobin CA (2012). STAR: ultrafast universal RNA-seq aligner. Bioinformatics.

[65] Anders S (2015). HTSeq- a Python framework to work with high-throughput sequencing data. Bioinformatics.

[66] Andrews, S. FastQC: A Quality Control Tool for High Throughput Sequence Data [Online]. Available online at: http://www.bioinformatics.babraham.ac.uk/projects/fastqc/ (2010).

[67] Love MI (2014). Moderated estimation of fold change and dispersion for RNA-seq data with DESeq2. Genome Biol..

[68] McAuliffe PF (2015). Ability to generate patient-derived breast cancer xenografts is enhanced in chemoresistant disease and predicts poor patient outcomes. PLoS One.

