# Supplementary material for: Epigenetically upregulating TROP2 and SLFN11 enhances therapeutic efficacy of TROP2 antibody drug conjugate sacitizumab govitecan
Source: NPJ Breast Cancer. 2023 Aug 11;9:66. doi: 10.1038/s41523-023-00573-8 (PMC10421911; doi:10.1038/s41523-023-00573-8)
Supplement: Supplementary file 2 — Supplementary Materials [file 41523_2023_573_MOESM2_ESM.pptx]

## Slide 1
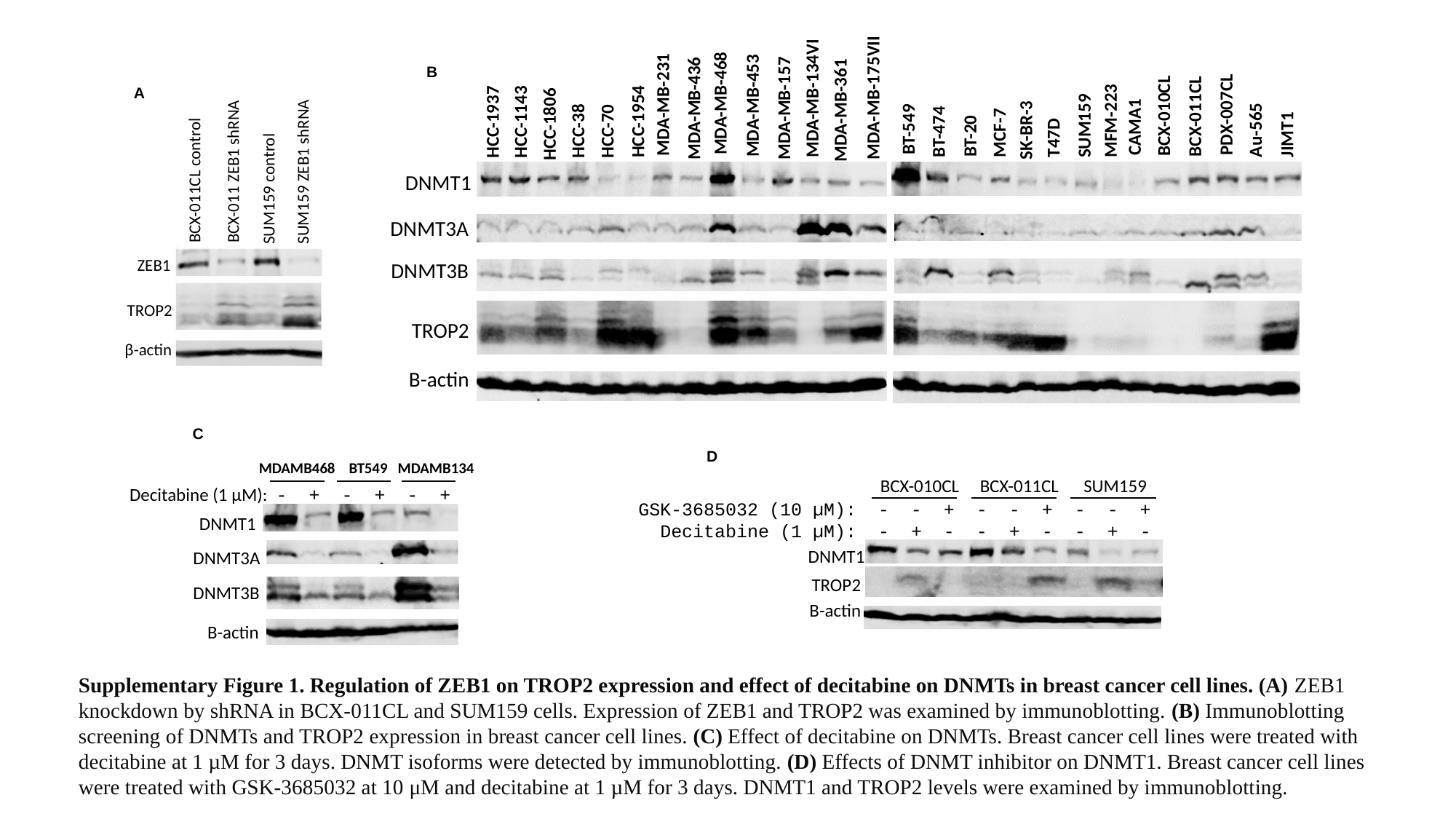

MDA-MB-175VII
MDA-MB-134VI
MDA-MB-468
MDA-MB-231
MDA-MB-453
MDA-MB-436
MDA-MB-157
MDA-MB-361
PDX-007CL
BCX-010CL
BCX-011CL
MFM-223
HCC-1954
HCC-1937
HCC-1143
HCC-1806
SUM159
CAMA1
BT-549
Au-565
SK-BR-3
HCC-38
HCC-70
BT-474
MCF-7
JIMT1
BT-20
T47D
DNMT1
DNMT3A
DNMT3B
TROP2
Β-actin
B
A
BCX-011 ZEB1 shRNA
SUM159 ZEB1 shRNA
BCX-011CL control
SUM159 control
ZEB1
TROP2
β-actin
C
D
MDAMB468 BT549 MDAMB134
Decitabine (1 µM): - + - + - +
DNMT1
DNMT3A
DNMT3B
Β-actin
BCX-010CL BCX-011CL SUM159
GSK-3685032 (10 µM): - - + - - + - - +
 Decitabine (1 µM): - + - - + - - + -
DNMT1
TROP2
Β-actin
Supplementary Figure 1. Regulation of ZEB1 on TROP2 expression and effect of decitabine on DNMTs in breast cancer cell lines. (A) ZEB1 knockdown by shRNA in BCX-011CL and SUM159 cells. Expression of ZEB1 and TROP2 was examined by immunoblotting. (B) Immunoblotting screening of DNMTs and TROP2 expression in breast cancer cell lines. (C) Effect of decitabine on DNMTs. Breast cancer cell lines were treated with decitabine at 1 µM for 3 days. DNMT isoforms were detected by immunoblotting. (D) Effects of DNMT inhibitor on DNMT1. Breast cancer cell lines were treated with GSK-3685032 at 10 μM and decitabine at 1 µM for 3 days. DNMT1 and TROP2 levels were examined by immunoblotting.

## Slide 2
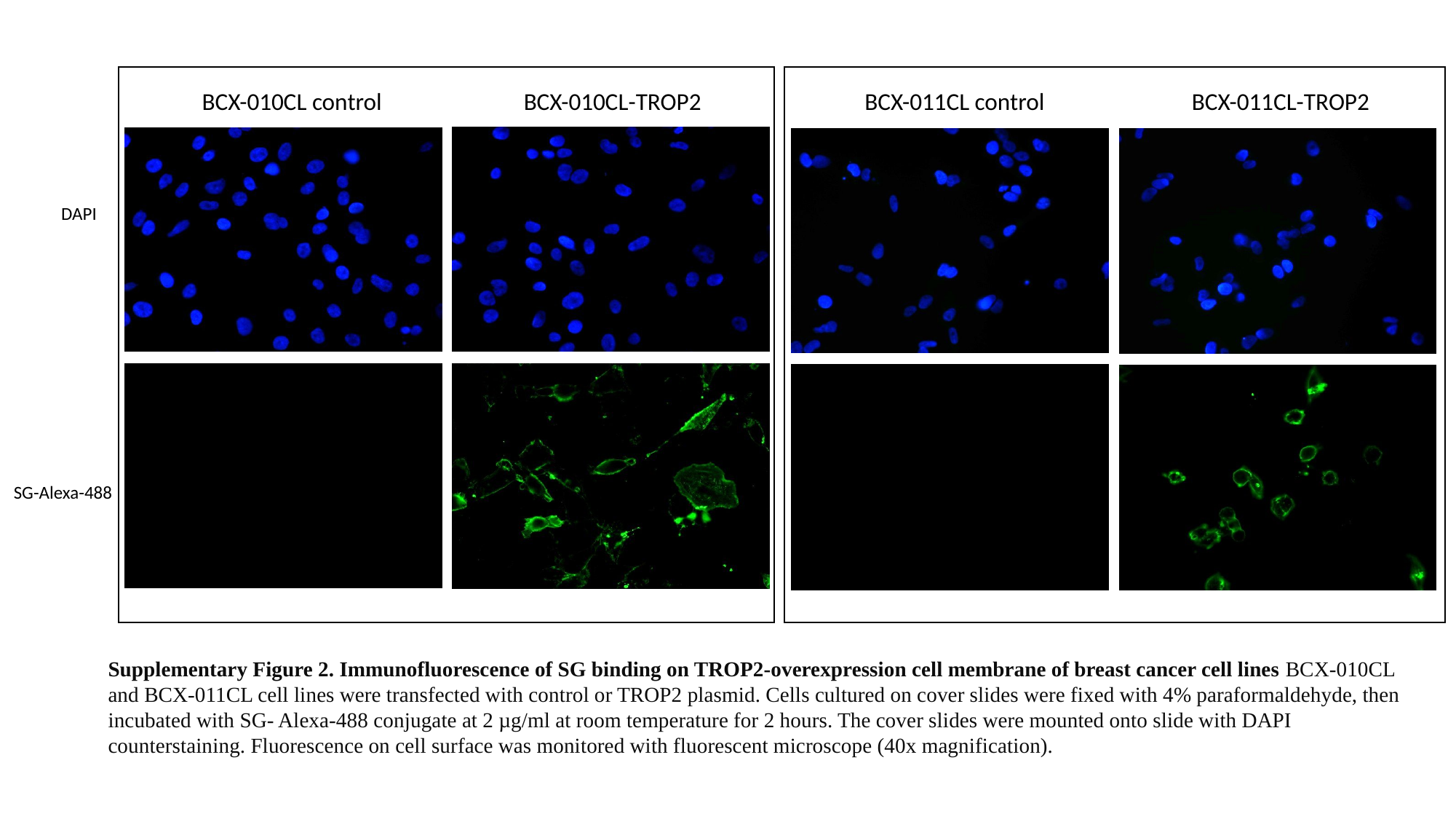

BCX-010CL control BCX-010CL-TROP2 BCX-011CL control BCX-011CL-TROP2
DAPI
SG-Alexa-488
Supplementary Figure 2. Immunofluorescence of SG binding on TROP2-overexpression cell membrane of breast cancer cell lines BCX-010CL and BCX-011CL cell lines were transfected with control or TROP2 plasmid. Cells cultured on cover slides were fixed with 4% paraformaldehyde, then incubated with SG- Alexa-488 conjugate at 2 µg/ml at room temperature for 2 hours. The cover slides were mounted onto slide with DAPI counterstaining. Fluorescence on cell surface was monitored with fluorescent microscope (40x magnification).

## Slide 3
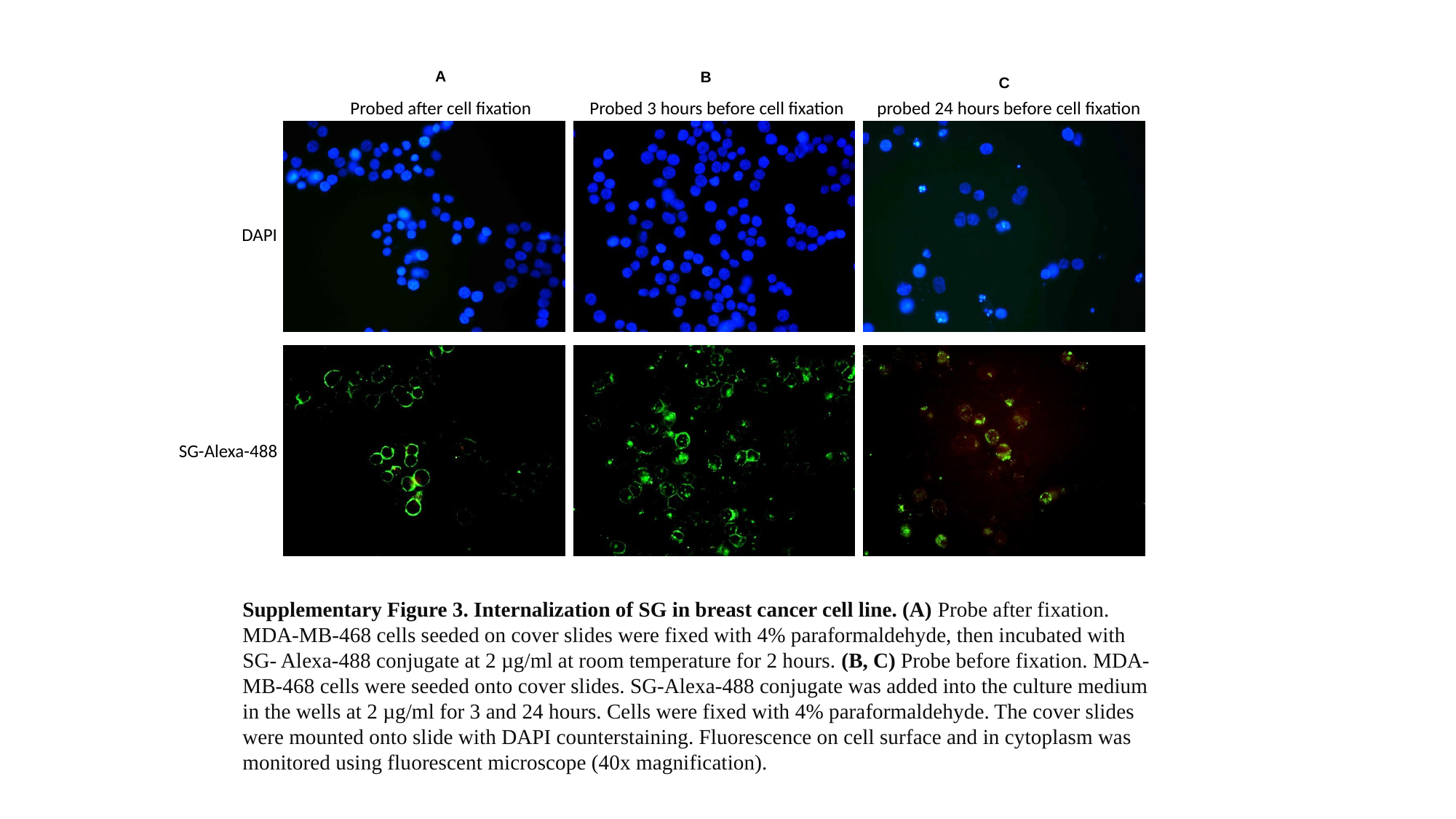

A
B
C
Probed after cell fixation Probed 3 hours before cell fixation probed 24 hours before cell fixation
DAPI
SG-Alexa-488
Supplementary Figure 3. Internalization of SG in breast cancer cell line. (A) Probe after fixation. MDA-MB-468 cells seeded on cover slides were fixed with 4% paraformaldehyde, then incubated with SG- Alexa-488 conjugate at 2 µg/ml at room temperature for 2 hours. (B, C) Probe before fixation. MDA-MB-468 cells were seeded onto cover slides. SG-Alexa-488 conjugate was added into the culture medium in the wells at 2 µg/ml for 3 and 24 hours. Cells were fixed with 4% paraformaldehyde. The cover slides were mounted onto slide with DAPI counterstaining. Fluorescence on cell surface and in cytoplasm was monitored using fluorescent microscope (40x magnification).

## Slide 4
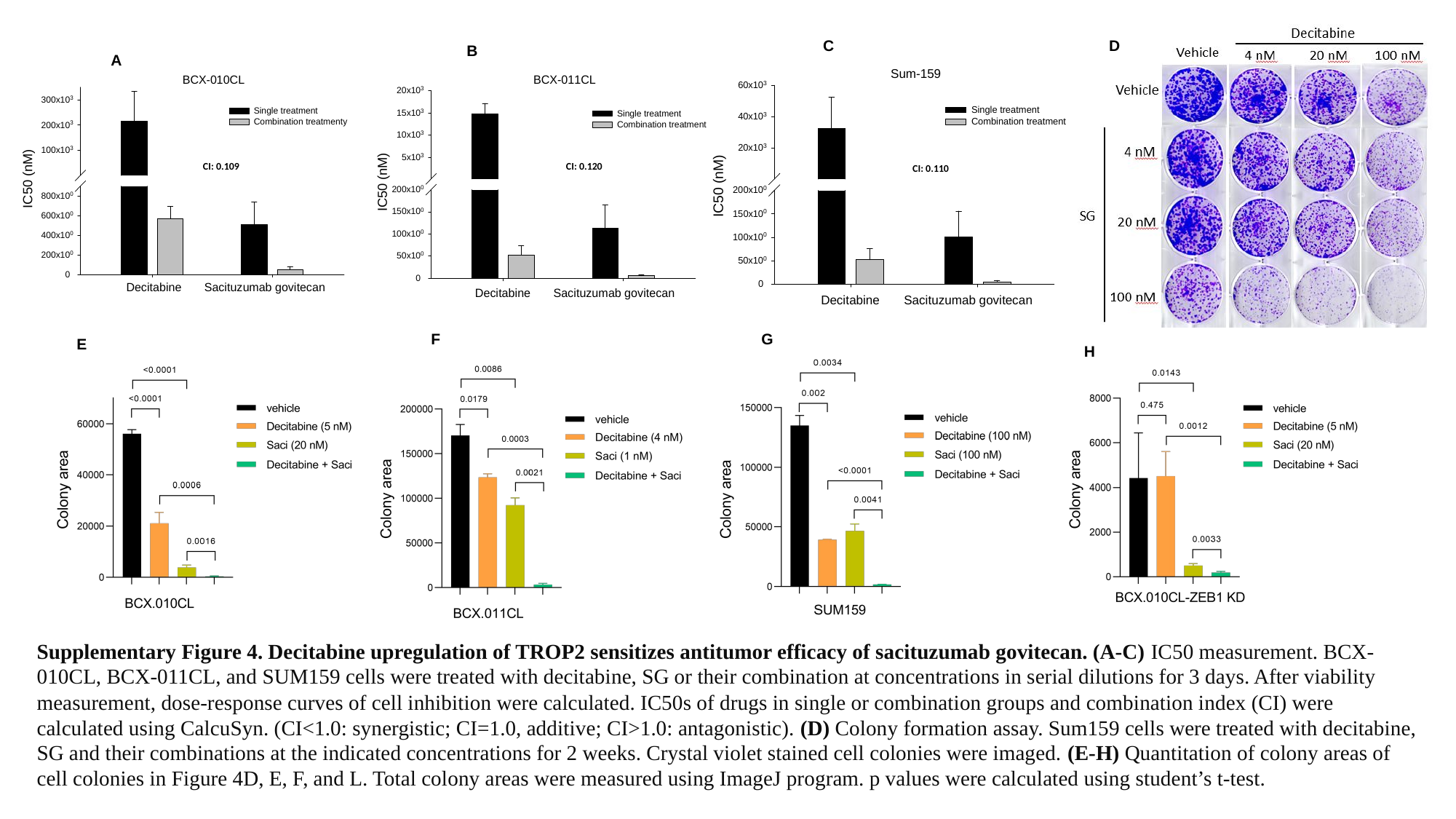

C
D
B
A
CI: 0.109
CI: 0.120
CI: 0.110
F
G
E
H
Supplementary Figure 4. Decitabine upregulation of TROP2 sensitizes antitumor efficacy of sacituzumab govitecan. (A-C) IC50 measurement. BCX-010CL, BCX-011CL, and SUM159 cells were treated with decitabine, SG or their combination at concentrations in serial dilutions for 3 days. After viability measurement, dose-response curves of cell inhibition were calculated. IC50s of drugs in single or combination groups and combination index (CI) were calculated using CalcuSyn. (CI<1.0: synergistic; CI=1.0, additive; CI>1.0: antagonistic). (D) Colony formation assay. Sum159 cells were treated with decitabine, SG and their combinations at the indicated concentrations for 2 weeks. Crystal violet stained cell colonies were imaged. (E-H) Quantitation of colony areas of cell colonies in Figure 4D, E, F, and L. Total colony areas were measured using ImageJ program. p values were calculated using student’s t-test.

## Slide 5
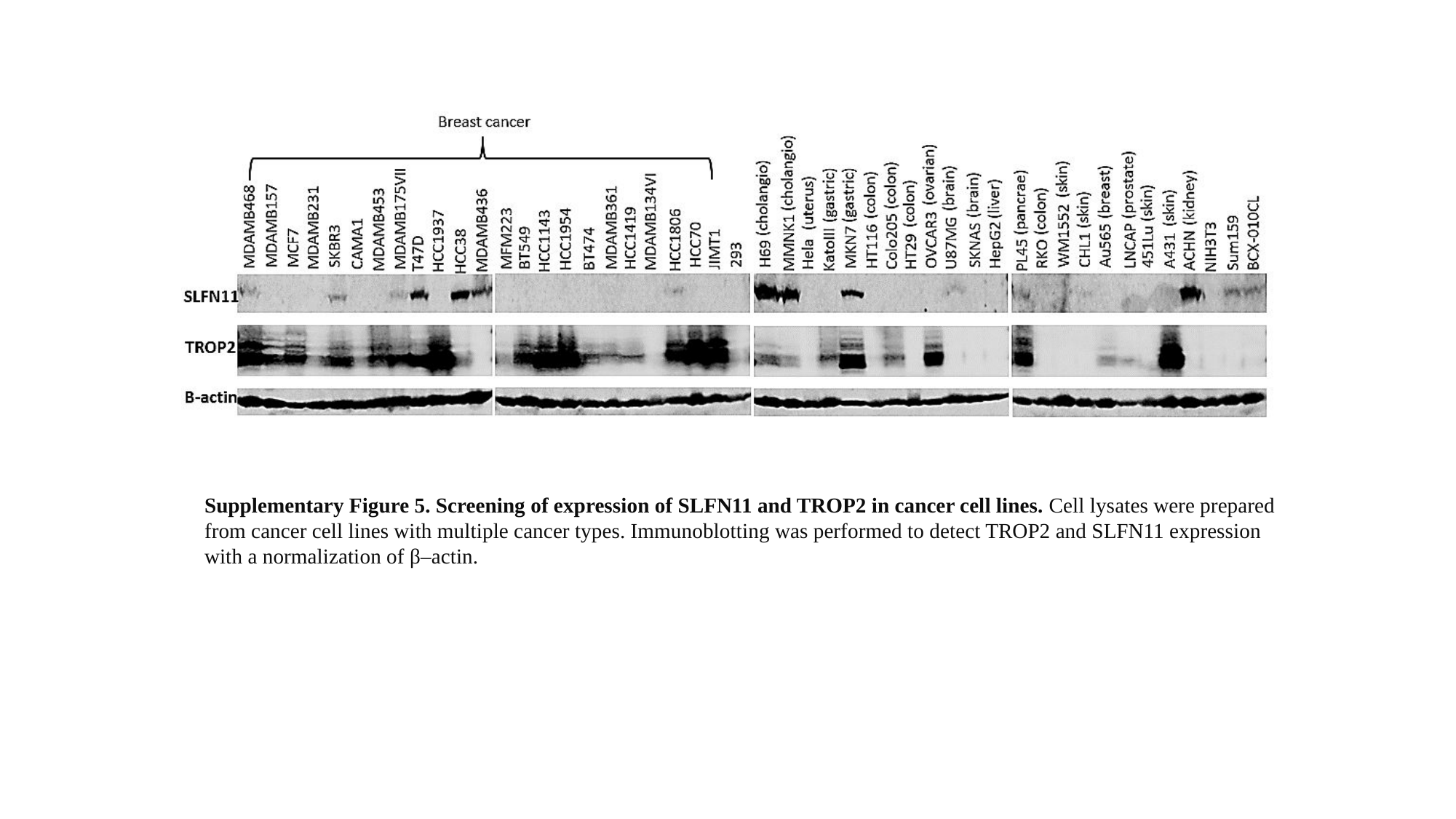

Supplementary Figure 5. Screening of expression of SLFN11 and TROP2 in cancer cell lines. Cell lysates were prepared from cancer cell lines with multiple cancer types. Immunoblotting was performed to detect TROP2 and SLFN11 expression with a normalization of β–actin.

## Slide 6
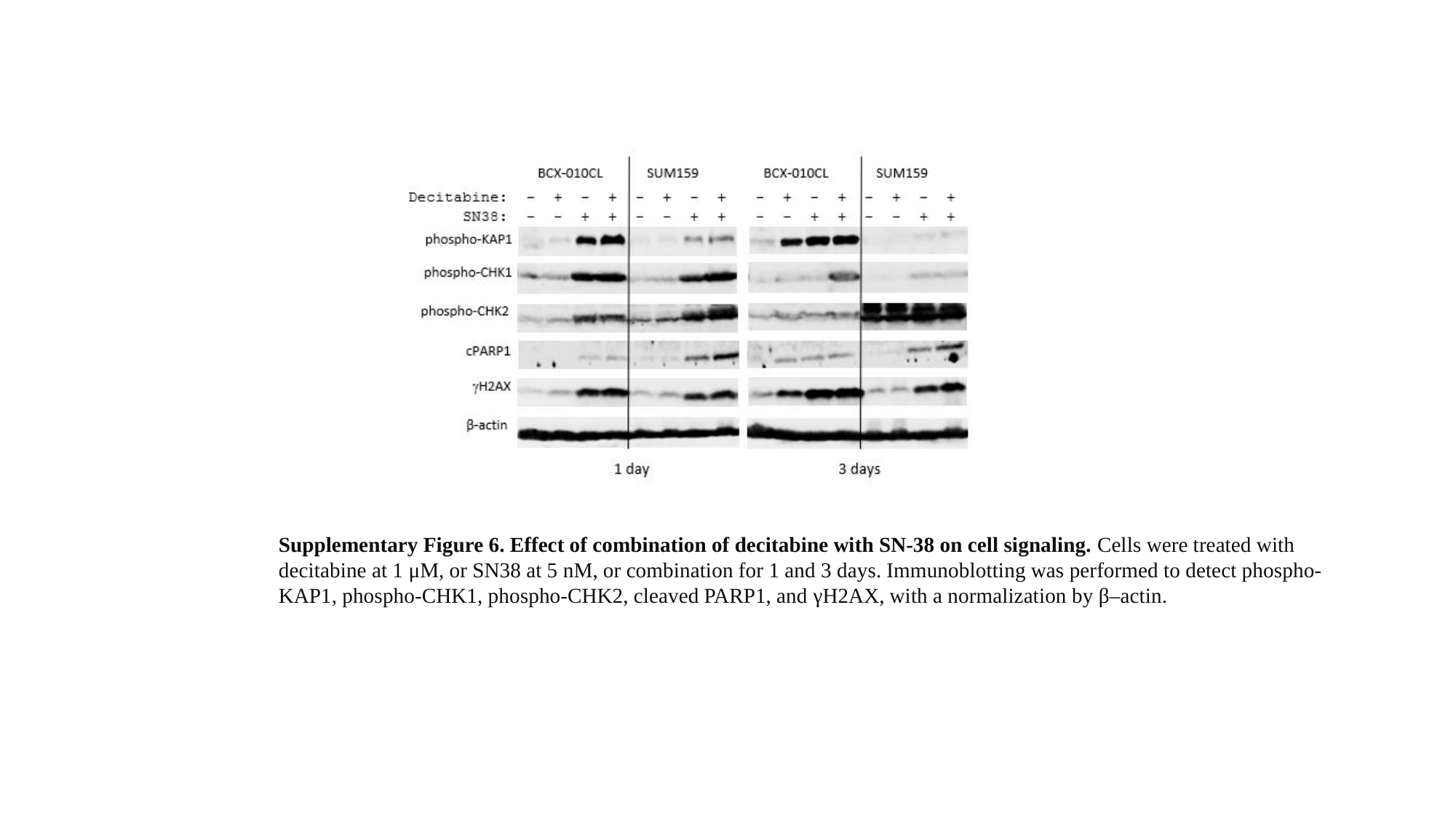

Supplementary Figure 6. Effect of combination of decitabine with SN-38 on cell signaling. Cells were treated with decitabine at 1 μM, or SN38 at 5 nM, or combination for 1 and 3 days. Immunoblotting was performed to detect phospho-KAP1, phospho-CHK1, phospho-CHK2, cleaved PARP1, and γH2AX, with a normalization by β–actin.

## Slide 7
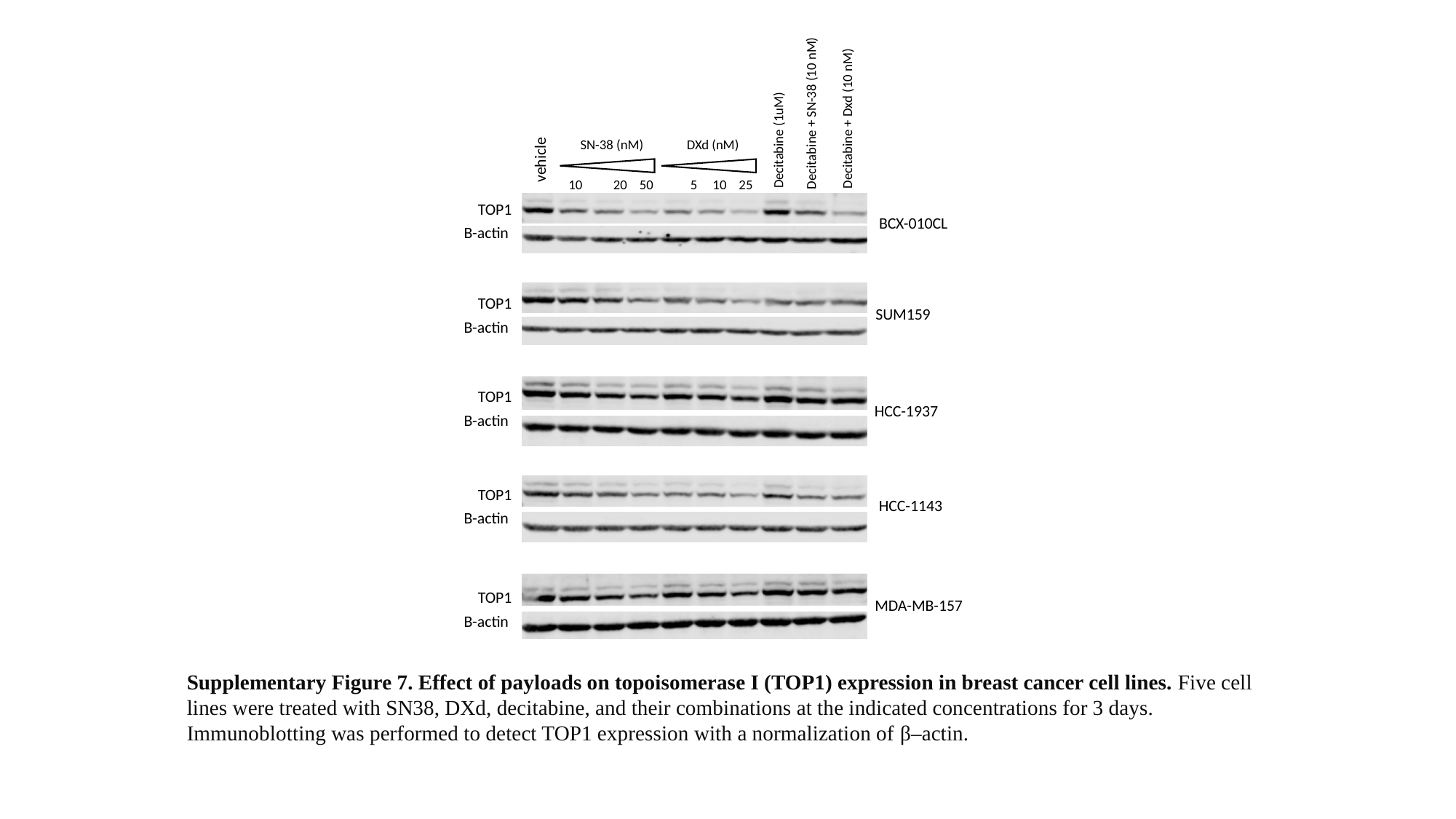

Decitabine + SN-38 (10 nM)
Decitabine + Dxd (10 nM)
Decitabine (1uM)
SN-38 (nM) DXd (nM)
vehicle
10 20 50 5 10 25
TOP1
BCX-010CL
Β-actin
TOP1
SUM159
Β-actin
TOP1
HCC-1937
Β-actin
TOP1
HCC-1143
Β-actin
TOP1
MDA-MB-157
Β-actin
Supplementary Figure 7. Effect of payloads on topoisomerase I (TOP1) expression in breast cancer cell lines. Five cell lines were treated with SN38, DXd, decitabine, and their combinations at the indicated concentrations for 3 days. Immunoblotting was performed to detect TOP1 expression with a normalization of β–actin.

## Slide 8
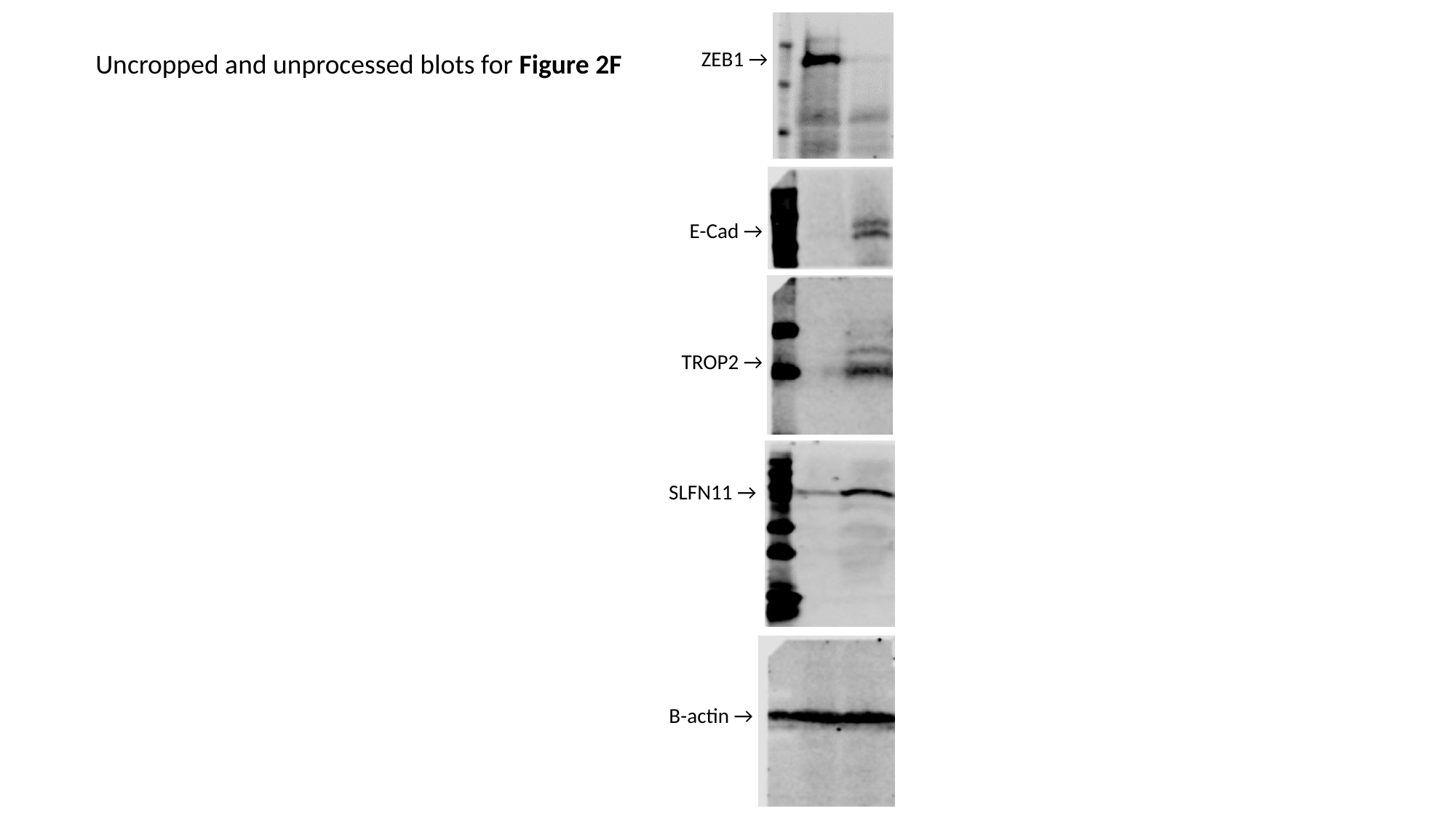

ZEB1 →
Uncropped and unprocessed blots for Figure 2F
E-Cad →
TROP2 →
SLFN11 →
Β-actin →

## Slide 9
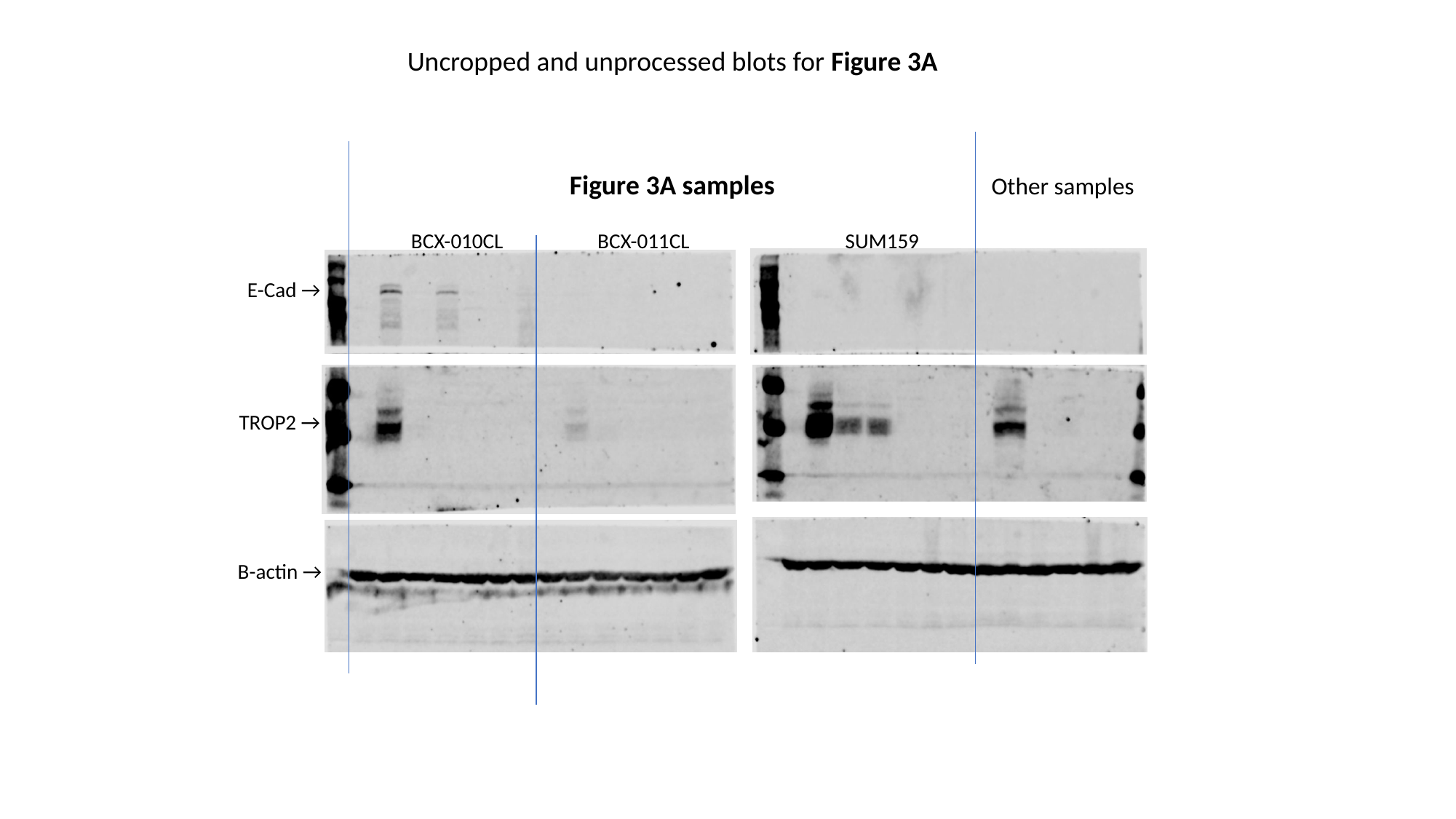

Uncropped and unprocessed blots for Figure 3A
Figure 3A samples Other samples
BCX-010CL BCX-011CL SUM159
E-Cad →
TROP2 →
Β-actin →

## Slide 10
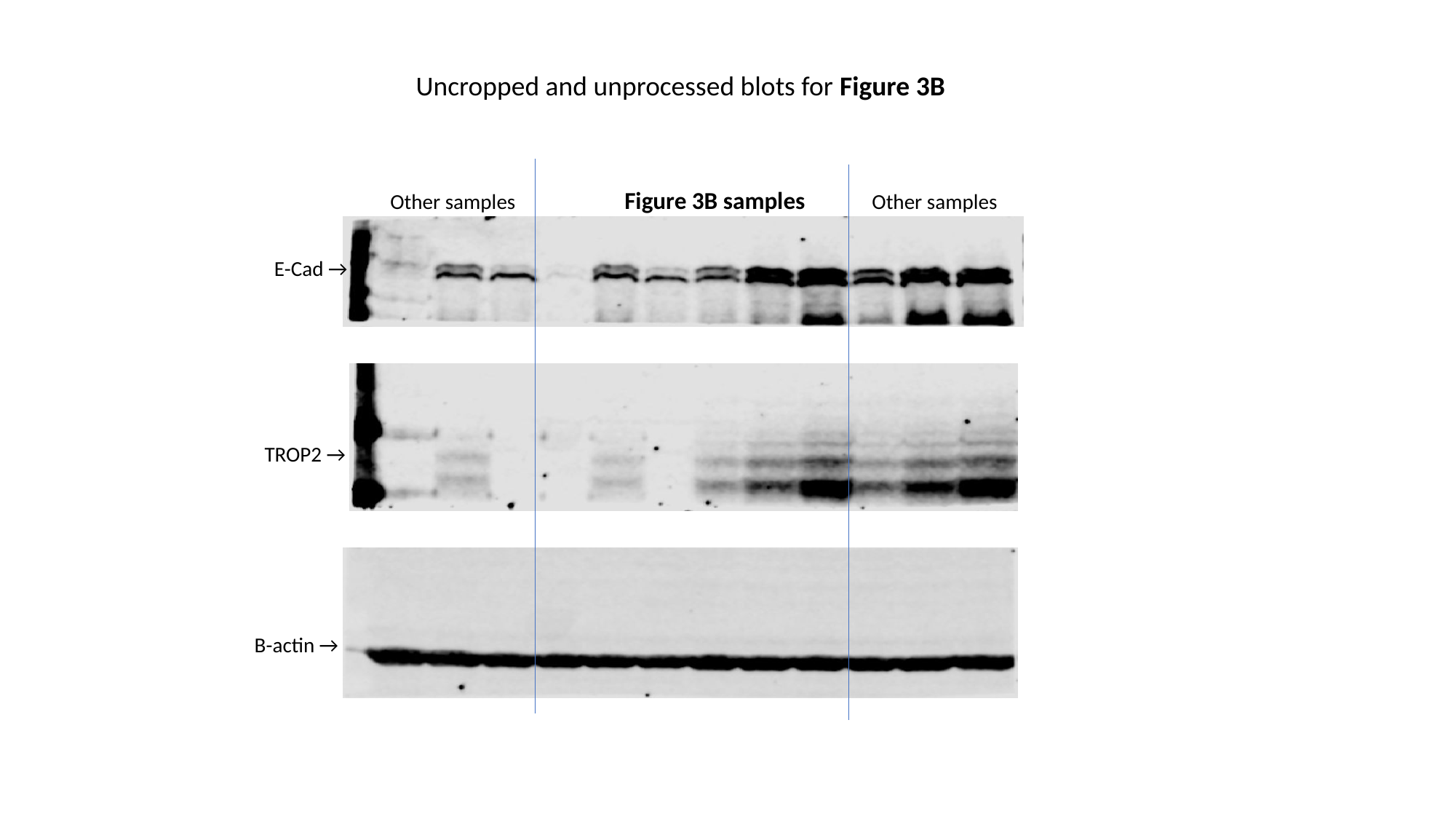

Uncropped and unprocessed blots for Figure 3B
Other samples Figure 3B samples Other samples
E-Cad →
TROP2 →
Β-actin →

## Slide 11
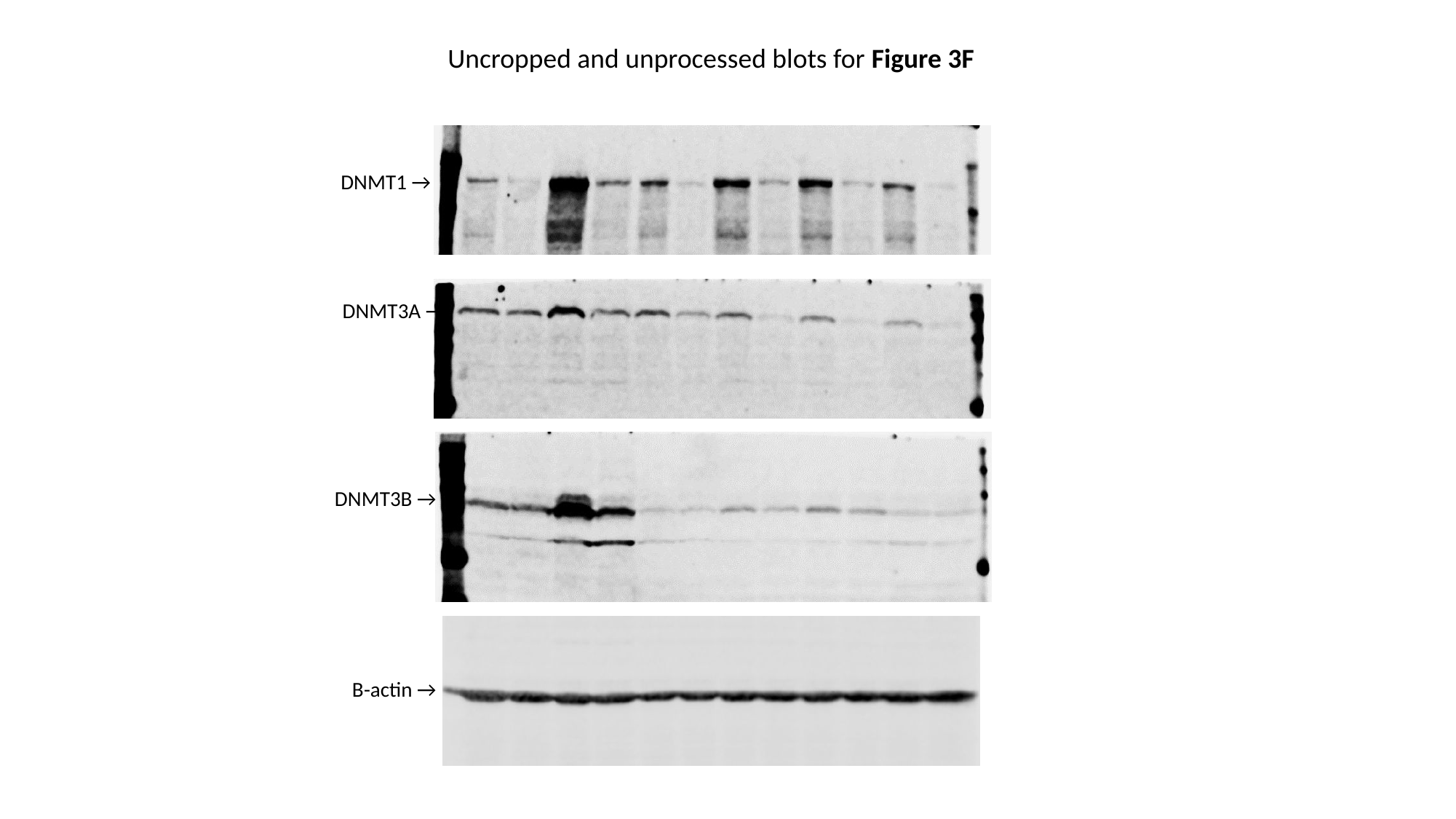

Uncropped and unprocessed blots for Figure 3F
DNMT1 →
DNMT3A →
DNMT3B →
Β-actin →

## Slide 12
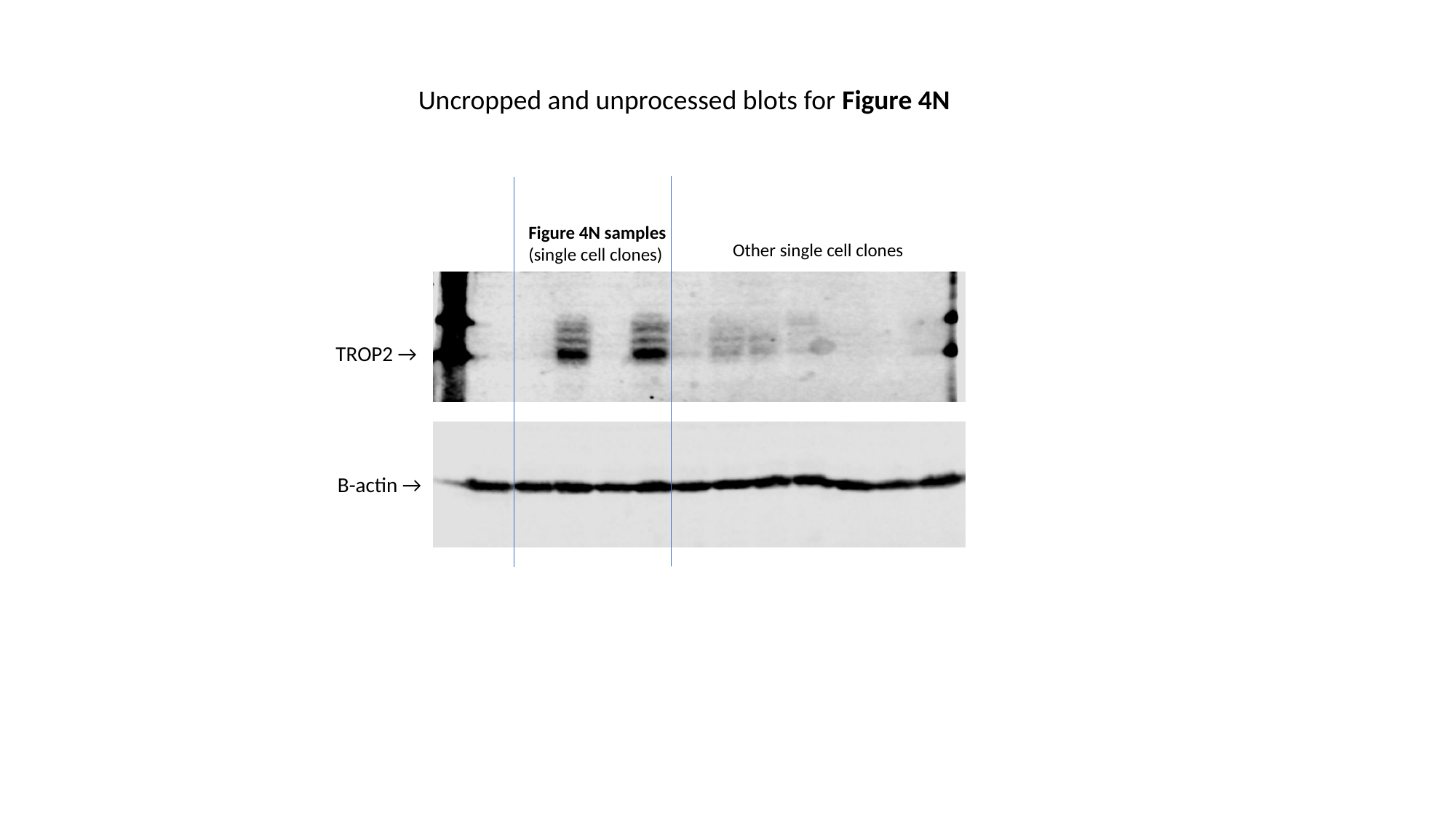

Uncropped and unprocessed blots for Figure 4N
Figure 4N samples
(single cell clones)
Other single cell clones
TROP2 →
Β-actin →

## Slide 13
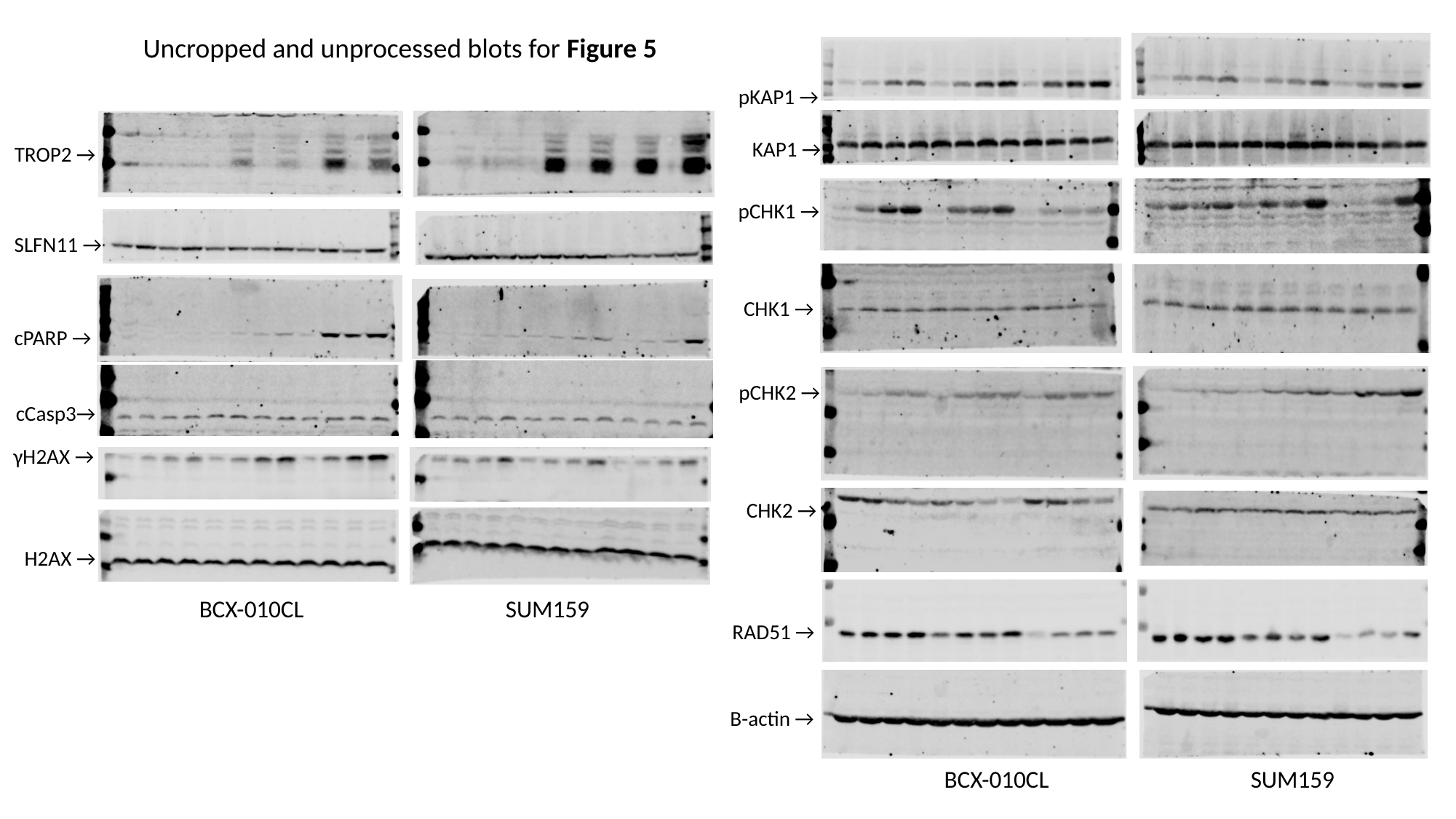

Uncropped and unprocessed blots for Figure 5
pKAP1 →
KAP1 →
TROP2 →
pCHK1 →
CHK1 →
SLFN11 →
cPARP →
pCHK2 →
CHK2 →
cCasp3→
γH2AX →
H2AX →
BCX-010CL SUM159
RAD51 →
Β-actin →
BCX-010CL SUM159

## Slide 14
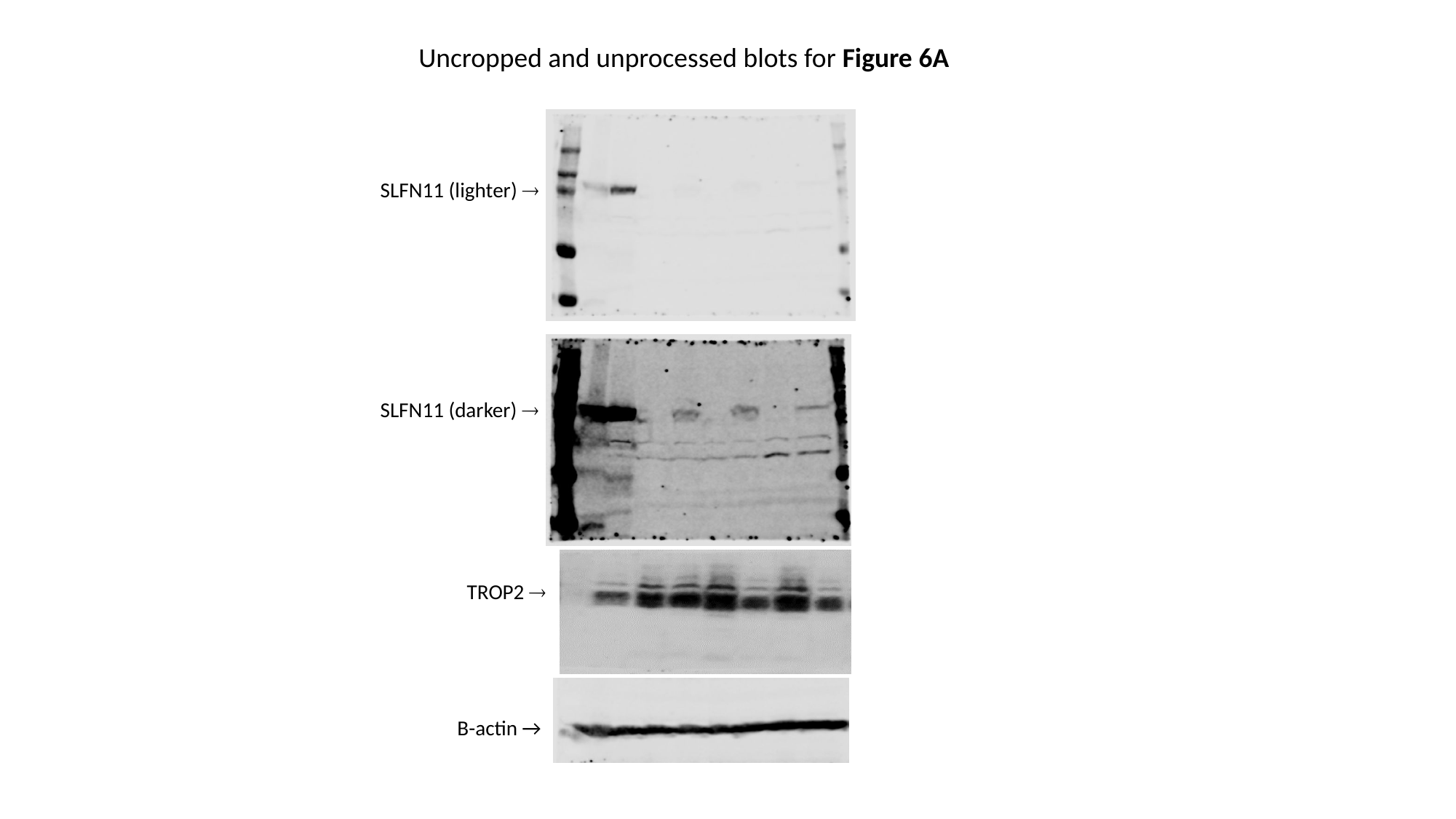

Uncropped and unprocessed blots for Figure 6A
SLFN11 (lighter) 
SLFN11 (darker) 
TROP2 
Β-actin →

## Slide 15
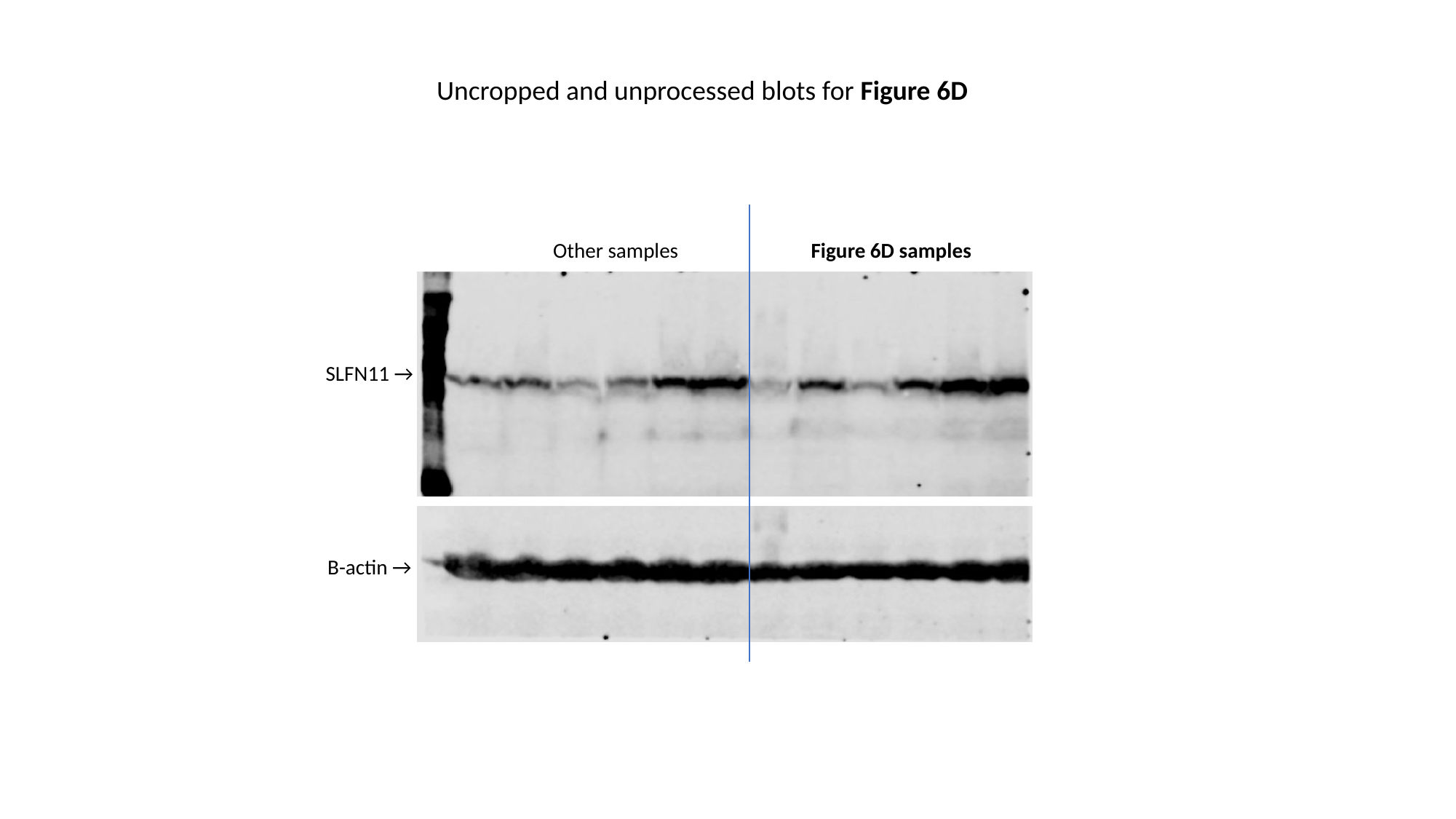

Uncropped and unprocessed blots for Figure 6D
Other samples Figure 6D samples
SLFN11 →
Β-actin →
